# Inhibition of Avian Influenza A Virus Replication in Human Cells by Host Restriction Factor TUFM Is Correlated with Autophagy

**DOI:** 10.1128/mBio.00481-17

**Published:** 2017-06-13

**Authors:** Shu-Ming Kuo, Chi-Jene Chen, Shih-Cheng Chang, Tzu-Jou Liu, Yi-Hsiang Chen, Sheng-Yu Huang, Shin-Ru Shih

**Affiliations:** a Research Center for Emerging Viral Infections, Chang Gung University, Taoyuan, Taiwan; b Department of Medical Biotechnology and Laboratory Science, College of Medicine, Chang Gung University, Taoyuan, Taiwan; c Clinical Virology Laboratory, Department of Laboratory Medicine, Chang Gung Memorial Hospital, Taoyuan, Taiwan; d Graduate Institute of Biomedical Sciences, Chang Gung University, Taoyuan, Taiwan; Mailman School of Public Health, Columbia University

**Keywords:** PB2, TUFM, autophagy, host restriction factor, influenza A virus, mitochondria, virus-host interactions

## Abstract

Avian influenza A viruses generally do not replicate efficiently in human cells, but substitution of glutamic acid (Glu, E) for lysine (Lys, K) at residue 627 of avian influenza virus polymerase basic protein 2 (PB2) can serve to overcome host restriction and facilitate human infectivity. Although PB2 residue 627 is regarded as a species-specific signature of influenza A viruses, host restriction factors associated with PB2_627_E have yet to be fully investigated. We conducted immunoprecipitation, followed by differential proteomic analysis, to identify proteins associating with PB2_627_K (human signature) and PB2_627_E (avian signature) of influenza A/WSN/1933(H1N1) virus, and the results indicated that Tu elongation factor, mitochondrial (TUFM), had a higher binding affinity for PB2_627_E than PB2_627_K in transfected human cells. Stronger binding of TUFM to avian-signature PB2_590_G/_591_Q and PB2_627_E in the 2009 swine-origin pandemic H1N1 and 2013 avian-origin H7N9 influenza A viruses was similarly observed. Viruses carrying avian-signature PB2_627_E demonstrated increased replication in TUFM-deficient cells, but viral replication decreased in cells overexpressing TUFM. Interestingly, the presence of TUFM specifically inhibited the replication of PB2_627_E viruses, but not PB2_627_K viruses. In addition, enhanced levels of interaction between TUFM and PB2_627_E were noted in the mitochondrial fraction of infected cells. Furthermore, TUFM-dependent autophagy was reduced in TUFM-deficient cells infected with PB2_627_E virus; however, autophagy remained consistent in PB2_627_K virus-infected cells. The results suggest that TUFM acts as a host restriction factor that impedes avian-signature influenza A virus replication in human cells in a manner that correlates with autophagy.

## IMPORTANCE

An understanding of the mechanisms that influenza A viruses utilize to shift host tropism and the identification of host restriction factors that can limit infection are both critical to the prevention and control of emerging viruses that cross species barriers to target new hosts. Using a proteomic approach, we revealed a novel role for TUFM as a host restriction factor that exerts an inhibitory effect on avian-signature PB2_627_E influenza virus propagation in human cells. We further found that increased TUFM-dependent autophagy correlates with the inhibitory effect on avian-signature influenza virus replication and may serve as a key intrinsic mechanism to restrict avian influenza virus infection in humans. These findings provide new insight regarding the TUFM mitochondrial protein and may have important implications for the development of novel antiviral strategies.

## INTRODUCTION

Seasonal influenza viruses circulating in human populations infect millions of people annually and have profound and costly public health consequences (1). In the 20th century, several influenza pandemics, such as those that occurred in 1918, 1957, and 1968, devastated human populations. Natural reservoirs of influenza A virus exist in wild waterfowl and domestic poultry (2), and the ability of such avian influenza A viruses to cross host barriers and ultimately adapt to humans is a key factor driving outbreaks. Most avian influenza A viruses are unable to infect humans, but those that have crossed the host barrier can cause serious infections (3); for example, an H5N1 influenza A virus highly pathogenic to both chickens and humans emerged in Hong Kong in 1997 and eventually killed 6 of 18 infected persons (4). Several fatal H5N1 influenza A virus outbreaks have occurred since then. In 2013, new avian-origin H7N9 influenza A viruses were reported in eastern China (5), currently yielding a fatality rate of >36.8% in humans (Food and Agriculture Organization. H7N9 situation update, 24 March 2017 [http://www.fao.org/ag/againfo/programmes/en/empres/h7n9/Situation_update.html]), on the basis of confirmed cases. These outbreaks underscore the need to understand how influenza A viruses cross species barriers and develop infectivity in humans.

Influenza A viruses are enveloped negative-stranded RNA viruses of the *Orthomyxoviridae* family that possess segmented genomes. Each ribonucleoprotein (RNP) complex of an influenza virion consists of an RNA strand packaged with four viral proteins, polymerase basic protein 1 (PB1), polymerase basic protein 2 (PB2), polymerase acid (PA) protein, and nucleoprotein (NP). The RNP complex drives viral replication in the host nucleus, enabling the virus to hijack host cell resources (6). A single substitution of glutamic acid (E, avian signature) for lysine (K, human signature) at residue 627 of PB2 is a major determinant for viruses to overcome host restrictions (7), as this substitution restores viral polymerase activity (8) and allows viruses to replicate efficiently in mammalian cells and animal models (9–11).

The host factors involved in the adaptive mechanism of PB2_627_ have been the focus of much research, and several hypotheses have emerged. In the first hypothesis, it is suggested that positive factors control the adaptive mechanism (12); for instance, importin-α1 and importin-α7 bind more strongly to the PB2_627_K-RNP complex, and this facilitates viral replication in human cells (13). Importin-α7 knockout (KO) mice are less susceptible to infection with viruses with PB2_627_K (13, 14), and thus, importin-α7 is considered to be a key positive factor. The second hypothesis posits that no restriction factors exist, but the decrease or disappearance of positive factors has an impact on the PB2_627_E-RNP complex (15). For example, for viruses carrying avian-signature PB2_627_E, chicken ANP32A enhances polymerase activity and viral replication in human cells to levels comparable to those of human-signature PB2_627_K, while human ANP32A lacks 33 key functional amino acids and therefore restricts the replication of avian influenza viruses in human cells (16). The third hypothesis posits that restriction factors selectively inhibit the avian-signature PB2_627_E-RNP complex in mammalian cells (17); for example, RIG-I has greater binding affinity than NP for PB2_627_E, and this disrupts the viral replication machinery in human cells (18); however, RIG-I knockdown failed to rescue PB2_627_E polymerase activity, suggesting that other restriction factors remain to be identified.

Although PB2 is localized primarily in the host nucleus and most PB2-interacting human proteins are nucleus related, PB2 signals have also been detected in the mitochondria, and a mitochondrial-targeting signal is present at the N terminus of PB2 (19, 20). Previous research has reported that PB2 can interact with the mitochondrial antiviral signaling (MAVS) protein to disrupt type I interferon (IFN) induction (21). It is possible that host mitochondrial factors can also interact with PB2 to disrupt viral adaptive mechanisms, and in this study, we found that Tu elongation factor, mitochondrial (TUFM, also known as EF-Tu, P43, or COXPD4), can act as a selective PB2_627_E restriction factor. TUFM is a fundamental mitochondrial protein that has been implicated in protein translation, GTPase activity, and RNA binding (22), and it has also been reported to act as an NLRX1-interacting partner that enhances autophagy while inhibiting MAVS protein-induced IFN-β expression in vesicular stomatitis virus (VSV)-infected cells (23). Host defense against microbes and viruses, including influenza A virus, is known to be one of the triggers of autophagy (24, 25). Influenza A viruses have also been shown to reduce autophagy via direct interactions between viral matrix 2 protein and the microtubule-associated protein 1 light chain 3 (LC3) autophagic protein (26).

Our previous research on species-associated genomic signatures sought to delineate the genetic boundary between avian and human influenza A viruses (27). In this study, we identified TUFM, a novel host restriction factor that demonstrated a higher binding affinity for avian-signature PB2_627_E than for human-signature PB2_627_K and that selectively inhibited PB2_627_E viral replication in human cells, potentially through the mediation of cellular autophagy.

## RESULTS

### Proteomic analysis of differential interactions with human cellular proteins for influenza A/WSN/1933(H1N1) virus PB2_627_K and PB2_627_E.

**FIG 1:**
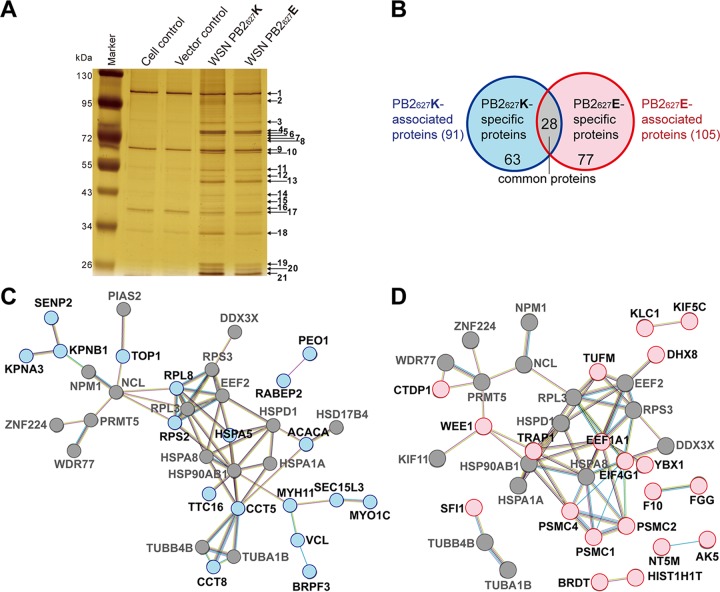
Proteomic profiling of cellular proteins associating with the WSN PB2_627_K and PB2_627_E proteins of influenza A virus. (A) Cellular proteins of human 293T cells associating with either PB2_627_K or PB2_627_E were purified and separated, and numbered protein bands were analyzed by MALDI-TOF MS (for the results, see Table S1). (B) Venn diagram of proteins associated with PB2_627_K and PB2_627_E. Each number is the number of proteins in each category. (C, D) Protein-protein interaction networks of PB2_627_K-associated (C) and PB2_627_E-associated (D) proteins. Common proteins are shown as gray nodes, while PB2_627_K- and PB2_627_E-specific proteins are shown as light blue and pink nodes, respectively. The different colors of the connecting lines represent the evidence backing each interaction, such as experiments (pink) and curated databases (cyan) for known interactions, gene fusion (red) for predicted interactions, coexpression (brown), and text mining (yellow). Interactions and connections with at least two proteins are presented.

To obtain differentially expressed PB2 constructs, FLAG-tagged PB2 of human influenza A/WSN/1933(H1N1) (WSN) virus was generated and named WSN PB2_627_K. A K627E substitution was then generated by site mutation, and the result was named WSN PB2_627_E. WSN PB2_627_E is defined as avian-like because its backbone was derived from human influenza A virus with only a single substitution in PB2 (K627E). Human proteins associated with WSN PB2_627_K and PB2_627_E were collected by FLAG immunoprecipitation (FLAG-IP), separated by gradient SDS-PAGE, subjected to silver staining (Fig. 1A), and identified by matrix-assisted laser desorption ionization (MALDI-TOF) mass spectrometry (MS) analysis. For detailed proteomic results see Table S1 in the supplemental material. A total of 168 putative associated proteins were identified, including 91 PB2_627_K-associated and 105 PB2_627_E-associated proteins. After 28 common proteins (gray) associated with both PB2 proteins were excluded, 63 PB2_627_K-specific and 77 PB2_627_E-specific proteins were classified (light blue and pink, respectively, in Fig. 1B).

10.1128/mBio.00481-17.9 TABLE S1  Results of proteomic analysis of cellular proteins interacting with WSN PB2_627_K and WSN PB2_627_E viral proteins in 293T cells. Download TABLE S1, XLS file, 0.2 MB. Copyright © 2017 Kuo et al. 2017 Kuo et al. This content is distributed under the terms of the Creative Commons Attribution 4.0 International license.

For details regarding the putative interacting proteins in each group that are enriched in biological processes, see Table S2. The protein-protein interactions show highly cohesive networks and reveal abundant functional interactions within networks (Fig. 1C and D). The results indicate that PB2_627_K- and PB2_627_E-associated proteins have different protein interaction profiles; however, interactions among nuclear proteins related to transcriptional regulatory functions, such as NCL, NPM1, PRMT5, ZNF224, and WDR77, are similar in both networks of PB2_627_K- and PB2_627_E-associated proteins. Interestingly, the interactions among the RPL3, RPS3, and EEF2 proteins in both networks are, respectively, connected by RPL8 and TUFM (Fig. 1C and D). TUFM is known to participate in various biological processes and possesses translation factor activity, translation elongation factor activity, GTPase activity, and nucleotide binding capability (see Table S2). Mitochondrial factors involved in influenza virus PB2_627_ adaptive mechanisms have not been well characterized to date, and therefore, TUFM was selected for further validation, as this study hypothesized that host restriction factors may interact exclusively with PB2_627_E to inhibit avian influenza virus replication in human cells.

10.1128/mBio.00481-17.10 TABLE S2  Enrichment analysis of biological processes for common, PB2_627_K-specific, and PB2_627_E-specific proteins in 293T cells. Download TABLE S2, TIF file, 0.4 MB. Copyright © 2017 Kuo et al. 2017 Kuo et al. This content is distributed under the terms of the Creative Commons Attribution 4.0 International license.

### Demonstration of higher binding affinity of TUFM for WSN PB2_627_E.

**FIG 2:**
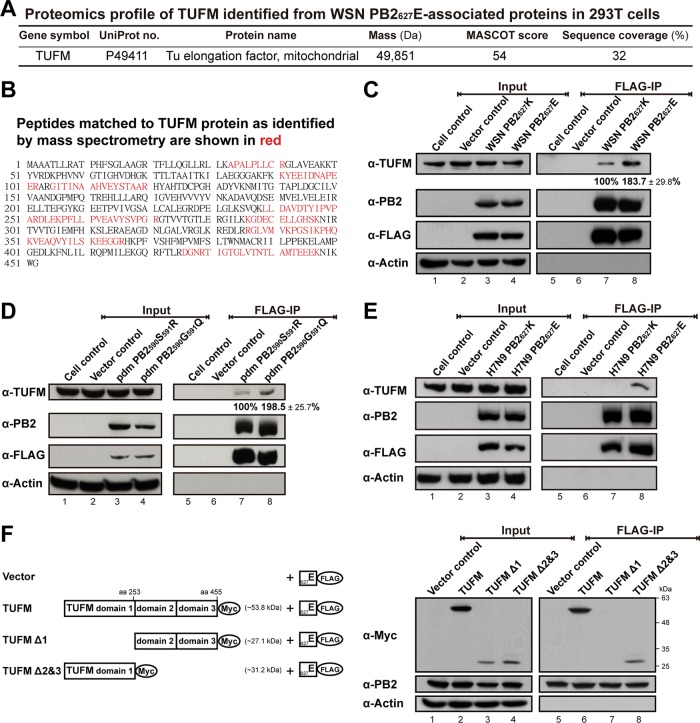
Higher binding affinity of TUFM for avian-signature PB2s of influenza A viruses in transfected human cells. (A) Proteomic profile of cellular TUFM identified from WSN PB2_627_E-associated proteins in 293T cells with the MASCOT database. (B) Peptides matched to TUFM protein, as identified by MS, are red. TUFM has a higher binding affinity for avian-signature PB2_627_E of A/WSN/1933(H1N1) (C), PB2_590_G/_591_Q of A/Taiwan/126/2009(pdmH1N1) (D), and PB2_627_E of A/Anhui/1/2013(H7N9) (E) influenza A viruses. The data are representative of three independent experiments. Total cell lysates (2 mg) were assayed by FLAG-IP, with results shown at the right and input proteins (50 μg) shown at the left. α-PB2 and α-FLAG represent FLAG-tagged PB2 proteins. α-Actin represents an NC, as it was not found to interact with PB2 (see Table S1). TUFM protein bands were quantitatively measured by ImageJ software. (F) Domain mapping of TUFM. Domain structures of human TUFM and predicted molecular masses of wild-type and truncated proteins are illustrated on the left. Myc-tagged wild-type TUFM or truncated TUFM Δ1 or TUFM Δ2&3 was cotransfected into 293T cells with FLAG-tagged WSN PB2_627_E. Cells transfected with the empty vector were used as an NC. Cell extracts were immunoprecipitated by FLAG-IP, and then PB2_627_E associations with truncated TUFM proteins were probed with anti-Myc antibody, and the results are shown on the right.

The proteomic profile of host factor TUFM is presented in Fig. 2A, and MS-matched TUFM peptides are listed in Fig. 2B. Total cell lysates were harvested from PB2-transfected 293T cells, and Western blotting was performed after FLAG-IP. The results indicate that TUFM has higher binding affinity for avian-signature PB2_627_E than for human-signature PB2_627_K in transfected 293T cells (lanes 7 and 8 of Fig. 2C), thus validating the proteomic analysis results.

One key concern regarding these findings is whether a TUFM homolog exists in avian species, and if so, whether such an avian TUFM homolog would demonstrate similar binding affinity. We sought to address this question in the most extensively studied avian species to date (as well as the species often hardest hit by avian influenza outbreaks), the chicken (*Gallus gallus*). Previously, the *G. gallus* genome database (version 6.5; updated on 4 January 2016) contained partial nucleotide sequences listed as chicken TUFM (chTUFM; XM_015274224), but owing to insufficient nucleotide sequence information, the generation of constructs to enable chTUFM protein expression or small interfering RNA (siRNA) design was very difficult. We therefore resolved the complete coding sequence (CDS) of chTUFM (GenBank accession no. KY769204). For a detailed sequence analysis of the key domains in human TUFM and its homologs in chickens and other species, see Fig. S1. We further investigated the binding affinity of chTUFM for WSN PB2_627_E in avian cells. After successfully constructing full-length chTUFM with a Myc tag expression plasmid, we performed Myc immunoprecipitation (Myc-IP) to compare the binding affinity of chTUFM with WSN PB2_627_K or PB2_627_E in DF-1 cells. We found that chTUFM exhibited no associations with either WSN PB2_627_K or PB2_627_E (see lanes 6 and 7 of Fig. S2A), in contrast to results derived from cotransfected Myc-tagged human TUFM and WSN PB2_627_E plasmids (see lane 8 of Fig. S2A) in DF-1 cells. From sequence alignment and phylogenetic analysis of TUFM homologs (see Fig. S1), it was observed that TUFM homologs in avian species appear to have undergone extensive evolution compared to mammalian species (see Fig. S1B to D). In addition, substitutions of 20, 7, and 3 amino acids (aa) were, respectively, noted in domain 1 (D1), D2, and D3 of avian TUFM compared with mammalian TUFM (see Fig. S1B), suggesting that avian TUFM may be fundamentally different from mammalian TUFM, and thus, chTUFM may have less affinity for the WSN PB2_627_ binding site (see Fig. S2A).

10.1128/mBio.00481-17.1 FIG S1  Bioinformatic analysis of TUFM homologs. (A) TUFM domain structures. Human TUFM was set as the reference sequence for residue numbers corresponding to GTP binding D1 (green), D2 (pink), and D3 (light blue). Bioinformatic analysis of TUFM proteins revealed that TUFM homologs in avian species had undergone extensive evolution compared to mammalian species, on the basis of amino acid alignment (B), phylogenetic tree analysis (C), and protein identity (D). The human (GenBank accession no. NP_003312), goat (XP_013830176), pig (XP_003124563), alpaca (XP_006201303), lemur (XP_012614397), dog (XP_536924), horse (XP_001502276), mouse (NP_766333), bat (XP_008155924), seal (XP_006734022), camel (XP_006183135), rabbit (XP_002711924), chicken (P84172), and kiwi (*Apteryx australis mantelli*; XP_013810087) TUFM protein sequences were aligned and analyzed with Geneious R9 software (44). Consensuses with the reference sequence are represented by dots. (B) Sequence logo representing the summarized polymorphisms of avian and mammalian TUFM proteins. (C) The unrooted phylogenetic tree was generated by the neighbor-joining method. Download FIG S1, TIF file, 0.5 MB. Copyright © 2017 Kuo et al. 2017 Kuo et al. This content is distributed under the terms of the Creative Commons Attribution 4.0 International license.

10.1128/mBio.00481-17.2 FIG S2  (A) chTUFM had no associations with PB2 residue 627, as observed in Myc-IP immunoprecipitates of Myc-tagged chTUFM cotransfected with WSN PB2_627_K or PB2_627_E into DF-1 cells. DF-1 cells cotransfected with Myc-tagged human TUFM and WSN PB2_627_E served as a positive control (lanes 4 and 8). Cells transfected with the empty vector were used as an NC (lanes 1 and 5). (B) Domain mapping of chTUFM. Diagram 1 shows DF-1 cells cotransfected with Myc-tagged human TUFM and FLAG-tagged WSN PB2_627_E, which served as a positive control. The Myc-tagged wild-type (diagram 2) and Δ1 (diagram 3) and Δ2&3 (diagram 4) truncated forms of chTUFM were cotransfected into DF-1 cells with FLAG-tagged WSN PB2_627_E. Cell extracts were immunoprecipitated by FLAG-IP, and then PB2_627_E associations with truncated TUFM proteins were probed with anti-Myc antibody. Download FIG S2, TIF file, 0.7 MB. Copyright © 2017 Kuo et al. 2017 Kuo et al. This content is distributed under the terms of the Creative Commons Attribution 4.0 International license.

### Higher binding affinity of TUFM for avian-signature PB2s from the 2009 pdmH1N1 and 2013 H7N9 influenza viruses.

We further assessed whether TUFM also has higher binding affinity for avian-signature PB2s derived from the 2009 pandemic H1N1 (pdmH1N1) and 2013 H7N9 influenza A viruses. In the 2009 swine-origin pdmH1N1 influenza A viruses, the substitutions in PB2, G590S and Q591R, are known genomic signatures that facilitate escape from host restriction mechanisms in human cells (28). We found that TUFM similarly demonstrated higher binding affinity for avian-signature PB2_590_G/PB2_591_Q than for human-signature PB2_590_S/PB2_591_R in transfected human cells (lanes 7 and 8 of Fig. 2D). In 2013 avian-origin H7N9 viruses that crossed species barriers to infect humans, we previously discovered that human-signature PB2_627_K was crucially associated with human infectivity in such viruses as well (29). We therefore transfected FLAG-tagged PB2_627_K or PB2_627_E from influenza A/Anhui/1/2013(H7N9) virus into 293T cells, and TUFM was also found to have higher affinity for avian-signature PB2_627_E than for human-signature PB2_627_K (lanes 7 and 8 of Fig. 2E). These results indicate that TUFM favors binding with avian-signature PB2 over human-signature PB2 in the WSN, pdmH1N1, and H7N9 influenza A virus strains.

The PB2 D701N substitution is a key determinant of the host range of influenza A viruses, and our previous research found that the PB2 D701N substitution restored polymerase activity in H7N9 viruses that retained avian signature PB2_627_E (29). In this study, we also examined the relevance of this substitution to the binding affinity of TUFM and found that H7N9 wild-type (701D; see lane 7 of Fig. S3A; similar to lane 7 of Fig. 2E) and mutant (D701N) PB2 had no association with TUFM, in contrast to the PB2_627_E positive control (see lane 6 of Fig. S3A; similar to lane 8 of Fig. 2E). Protein modeling of the H7N9 PB2 C-terminal domain (CTD) further indicated that the position of 701 is not in close proximity to aa 627, 590, and 591 (see Fig. S3B), which are known to associate with TUFM (Fig. 2C to E). On the basis of these findings, interactions between TUFM and the PB2 701D/N site are considered to be unlikely.

10.1128/mBio.00481-17.3 FIG S3  (A) PB2 residue 701 did not affect the interaction with TUFM, as observed in FLAG-IP immunoprecipitates from cells transfected with FLAG-tagged PB2 wild-type 701D and mutant D701N from the influenza A/Anhui/1/2013(H7N9) virus. Cells cotransfected with FLAG-tagged H7N9 PB2_627_E and human TUFM served as a positive control (lanes 2 and 6). Cells transfected without H7N9 PB2 were used as an NC. (B) Modeling of the A/Anhui/1/2013(H7N9) virus PB2 CTD based on the A/Vietnam/1203/2004(H5N1) virus (PDB ID 3KC6). The distribution of electrostatic potential on the protein surface (blue, relative positive charge; red, relative negative charge) is indicated. Positions 590, 591, 627, and 701 are indicated. Download FIG S3, TIF file, 1.5 MB. Copyright © 2017 Kuo et al. 2017 Kuo et al. This content is distributed under the terms of the Creative Commons Attribution 4.0 International license.

### TUFM D1 is required for association with avian-signature PB2_627_E.

To better elucidate the interaction between TUFM and avian-signature PB2_627_E, we sought to identify the essential TUFM domain involved in the binding of these two proteins. TUFM D1, D2, and D3 and the corresponding start and end amino acid residues are shown in Fig. S1A. GTP-binding D1 was previously shown to be essential for Atg5-Atg12 conjugate recruitment (23), and therefore, we generated TUFM Δ1 (deletion of D1) and TUFM Δ2&3 (deletion of D2 and D3) truncations for further investigation. We found that the interaction between PB2_627_E and TUFM required D1, given that its deletion (TUFM Δ1) abolished this interaction (lane 7 of Fig. 2F); however, D2 and D3 did not appear to be as critical to the interaction (lane 8 of Fig. 2F). A similar assay was performed with wild-type and truncated chTUFM, but none of these proteins demonstrated any association with PB2_627_E (see lanes 6 to 8 of Fig. S2B), compared to a positive control with human TUFM (see lane 5 of Fig. S2B; similar to lane 6 of Fig. 2F). Sequence alignment comparison of avian TUFM and mammalian TUFM (see Fig. S1B) showed that extensive polymorphisms, including 20 aa substitutions, occurred in D1 (as known as GTP-binding D1), and thus, the lack of association between chTUFM and PB2_627_E may be due to these in sequence and structure differences in D1.

### TUFM inhibits avian-signature PB2_627_E viral replication in human cells.

**FIG 3:**
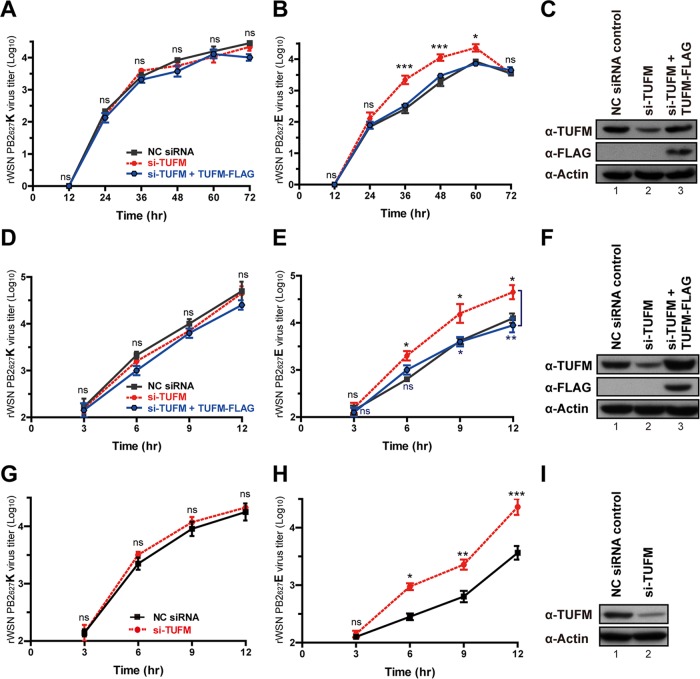
TUFM inhibits replication of influenza A virus with avian-signature PB2_627_E in infected human cells. (A, B) Growth kinetics of rWSN PB2_627_K (A) and PB2_627_E (B) viruses at an MOI of 0.001 for multicycle infections of human A549 cells transfected with an NC siRNA (black), si-TUFM (red), or si-TUFM plus TUFM-FLAG (blue) plasmid. (C) Transfection efficiency was assessed by Western blotting. (D, E) Growth kinetics of rWSN PB2_627_K (D) or PB2_627_E (E) virus at an MOI of 2 for a single infection cycle in human A549 cells. (F) Transfection efficiency assessed by Western blotting. (G, H) Growth kinetics of rWSN PB2_627_K (G) or PB2_627_E (H) virus at an MOI of 2 for a single infection cycle in NHBE cells transfected with NC siRNA (black) or si-TUFM (red). (I) Transfection efficiency assessed by Western blotting. Statistical analyses were conducted with GraphPad Prism 5. Data are the mean ± the standard error of the mean of three independent experiments. Statistical significance was determined by two-way analysis of variance. *, *P* < 0.05; **, *P* < 0.01; ***, *P* < 0.001; ns, no significance.

We sought to investigate the impact of TUFM on the replication of influenza A viruses in human cells. To obtain viruses carrying avian-signature PB2_627_E instead of PB2_627_K, reverse genetics was used to generate the K627E substitution of PB2 in the rWSN PB2_627_K virus to derive the rWSN PB2_627_E virus. Human A549 cells were used for initial viral growth kinetic studies, and A549 cells treated with TUFM siRNA (si-TUFM), si-TUFM plus the FLAG-tagged TUFM plasmid (TUFM-FLAG, for overexpression of TUFM), or a negative control (NC) were, respectively, infected with either rWSN PB2_627_K or PB2_627_E at a multiplicity of infection (MOI) of 0.001 or 2 (Fig. 3A to F). The growth curves of rWSN PB2_627_K virus showed no significant difference in A549 cells in either multicycle or single-cycle experiments (Fig. 3A and D), indicating that TUFM had no effect on rWSN PB2_627_K. Conversely, viral yields of rWSN PB2_627_E in TUFM-deficient (treated with si-TUFM) A549 cells significantly increased from 4.4- to 9.3-fold over controls at 36 to 60 h postinfection (hpi) with an MOI of 0.001 (Fig. 3B) and increased 5.0- to 5.9-fold over controls at 6 to 12 hpi with an MOI of 2 (Fig. 3E). Viral yields in A549 cells transfected with si-TUFM plus TUFM-FLAG were similar to controls (Fig. 3A, B, D, and E), indicating an absence of off-target effects with si-TUFM, while also confirming that rWSN PB2_627_E replication increases in TUFM-deficient A549 cells.

We further used normal human bronchial epithelial (NHBE) cells for investigation, but as NHBE cells became more fragile after siRNA transfection by nucleofection, only single-cycle experiments were conducted, and additional TUFM-FLAG plasmid transfections were not performed. Results showed that the viral growth kinetics of rWSN PB2_627_K at 3 to 12 hpi with an MOI of 2 were not affected in TUFM-deficient NHBE cells, but rWSN PB2_627_E viral yields significantly increased from 5.2- to 8.0-fold over controls at 6 to 12 hpi with an MOI of 2 (Fig. 3G to I). These results indicate that the presence of TUFM specifically impeded the replication of the avian-signature PB2_627_E virus but not that of the human-signature PB2_627_K virus.

A stable TUFM heterozygotic +/− KO MDCK cell line was generated with CRISPR/Cas9 endonuclease (see Fig. S4A), and it contained a second allele with a 2-nucleotide (nt) deletion in comparison with the wild type (see Fig. S4B). The deletion at nt 859 to 860 engendered a frameshift from aa 106 and ultimately resulted in a stop codon that ended translation at aa 114, as indicated by sequencing results (see Fig. S4C). Viral yields of rWSN PB2_627_E substantially increased from 12.4- to 14.5-fold over the wild type in the TUFM +/− KO MDCK cell line at 48 to 72 hpi with an MOI of 0.001 (see Fig. S4D), indicating that TUFM inhibits avian-signature PB2_627_E viral replication in canine cells. Protein levels of the stable TUFM +/− KO MDCK cell line were confirmed by Western blotting to be successfully knocked down (see Fig. S4E). No significant difference between the viability of TUFM-deficient human cells and TUFM +/− KO MDCK cells was observed in 3-(4,5-dimethyl-2-thiazolyl)-2,5-diphenyl-2H-tetrazolium bromide (MTT; Millipore) assays (see Fig. S4F).

10.1128/mBio.00481-17.4 FIG S4  TUFM inhibits replication of influenza A virus with avian-signature PB2_627_E in a TUFM heterozygotic +/− KO MDCK cell line established with the CRISPR/Cas9 system. (A) The sgRNA gene targets the third exon of the canine TUFM gene and contains a hexathymidine U6 gene termination signal. The canine TUFM gene that is the target for hybridization to 20 nt with the synthetic sgRNA molecule is illustrated in red. (B) TUFM knockdown in the expanded colonies was confirmed by F-PCR. (C) TUFM +/− KO MDCK cells presented two different alleles, as confirmed by TA cloning and sequencing. (D) Growth kinetics of rWSN PB2_627_E virus at an MOI of 0.001 for multicycle infections of wild-type (WT) and TUFM +/− KO MDCK cells. (E) Confirmation of TUFM protein expression in the TUFM +/− KO cell line by Western blotting. (F) Validation of viability of TUFM +/− KO MDCK cells with the MTT assay. Statistical analyses were conducted with GraphPad Prism 5. Data are the mean ± the standard error of the mean of three independent experiments. Statistical significance was determined by two-way analysis of variance for panel D and unpaired *t* tests for panel F. **, *P* < 0.01; ns, no significance. Download FIG S4, TIF file, 0.8 MB. Copyright © 2017 Kuo et al. 2017 Kuo et al. This content is distributed under the terms of the Creative Commons Attribution 4.0 International license.

TUFM-deficient A549 cells transfected with a TUFM-FLAG plasmid were found to have restricted viral replication compared to that of controls, as shown by viral titers (blue lines in Fig. 3B and E). However, wild-type A549 cells transfected with the TUFM-FLAG plasmid did not display lower viral titers than controls (see Fig. S5A to E), presumably because TUFM is already abundant in A549 cells. Growth curves of rWSN PB2_627_E viruses in different cell systems (see Fig. S5F to J) showed that higher viral titers could be seen in avian DF-1 cells (see Fig. S5G and I) than in human A549 cells (see Fig. S5B and D), indicating that avian-signature influenza A virus does not replicate efficiently in human cells.

10.1128/mBio.00481-17.5 FIG S5  Growth kinetics of rWSN PB2_627_K and PB2_627_E viruses in human and avian cells in which TUFM (A to J) and chTUFM (K to T) are exogenously overexpressed. Growth kinetics of rWSN PB2_627_K (A, C, F, H) and PB2_627_E (B, D, G, I) viruses at an MOI of 0.001 for multicycle infections (A, B, F, G) and an MOI of 2 for a single infection cycle (C, D, H, I) in human A549 cells (A to D; solid lines) and avian DF-1 cells (F to I; dashed lines) transfected with TUFM-FLAG (blue lines) and empty vector plasmids (gray lines). Overexpression efficiency of human TUFM in A549 (E) and DF-1 (J) cells was validated by Western blotting. Shown are the growth kinetics of rWSN PB2_627_K (K, M, P, R) and PB2_627_E (L, N, Q, S) viruses at an MOI of 0.001 (K, L, P, Q) and an MOI of 2 (M, N, R, S) in A549 (K to N; solid lines) and DF-1 (P to S; dashed lines) cells transfected with chTUFM-Myc (red lines) and empty vector plasmids (gray lines). Overexpression efficiency of chTUFM in A549 (O) and DF-1 (T) cells was validated by Western blotting. Statistical analyses were conducted with GraphPad Prism 5. Data are the mean ± the standard error of the mean of three independent experiments. Statistical significance was determined by two-way analysis of variance. ns, no significance. Download FIG S5, TIF file, 1.1 MB. Copyright © 2017 Kuo et al. 2017 Kuo et al. This content is distributed under the terms of the Creative Commons Attribution 4.0 International license.

We further sought to investigate the impact of chTUFM on the replication of both rWSN PB2_627_K and PB2_627_E viruses in chTUFM-deficient avian cells. The growth curves of both viruses showed no significant difference in either multicycle or single-cycle experiments conducted with chTUFM-deficient DF-1 cells (see Fig. S6), indicating that chTUFM had no effect on both viruses in avian cells. No significant differences were observed in the viability of chTUFM-deficient avian cells and control cells (see Fig. S6F). In addition, the growth curves of both viruses also showed no significant difference in human and avian cells exogenously expressing chTUFM (see Fig. S5K to T). Therefore, the inhibition of avian-signature PB2_627_E viral replication by TUFM appears to be specific for human cells (Fig. 3) but not chicken cells (see Fig. S6).

10.1128/mBio.00481-17.6 FIG S6  (A, B) Growth kinetics of rWSN PB2_627_K (A) and PB2_627_E (B) viruses at an MOI of 0.001 for multicycle infection of avian DF-1 cells transfected with NC siRNA (black), siRNA-1 of chTUFM (si-chTUFM-1, red), or siRNA-2 of chTUFM (si-chTUFM-2, blue). (C, D) Growth kinetics of rWSN PB2_627_K (C) or PB2_627_E (D) virus at an MOI of 2 for a single infection cycle in avian DF-1 cells transfected with NC siRNA (black), si-chTUFM-1 (red), or si-chTUFM-2 (blue). (E) chTUFM knockdown efficiency in DF-1 cells as assessed by qRT-PCR. Relative expression of chTUFM was normalized to chicken GAPDH. (F) Validation of viability of chTUFM-deficient avian DF-1 cells with the MTT assay. Statistical analyses were conducted with GraphPad Prism 5. Data are the mean ± the standard error of the mean of three independent experiments. Statistical significance was determined by two-way analysis of variance for panels A to D and by unpaired *t* tests for panels E and F. **, *P* < 0.01; ns, no significance. Download FIG S6, TIF file, 0.7 MB. Copyright © 2017 Kuo et al. 2017 Kuo et al. This content is distributed under the terms of the Creative Commons Attribution 4.0 International license.

### Colocalization and copurification of TUFM-PB2 in mitochondria

Since increased polymerase activity is one of the characteristics of viruses bearing the PB2 E627K and G590S/Q591R signatures and is also believed to be one of the key mechanisms that facilitate escape from host restriction mechanisms in humans, we sought to investigate the effects of TUFM on the polymerase activity in human cells. However, no significant difference in polymerase activities from both WSN PB2 627 K/E and pdmH1N1 PB2 590S/G-591R/Q RNP-NP complexes at both 37°C and 33°C in TUFM-deficient 293T cells was observed (see Fig. S7), although slight variations in the polymerase activity of WSN PB2_627_E at 37°C were noted (see Fig. S7A). We speculated that polymerase activity assays that mimic the viral RNP-NP complex in its nuclear functions may have a limited ability to measure the effect of mitochondrial proteins on viral replication, and therefore, we further investigated the interaction between TUFM and PB2 in mitochondria.

10.1128/mBio.00481-17.7 FIG S7  Effect of TUFM on the polymerase activity of the WSN PB2_627_K, PB2_627_E (A, B), pdmH1N1 PB2_590_S_591_R, or PB2_590_G_591_Q (C, D) RNP-NP complex at 37°C and 33°C in 293T cells treated with NC siRNA, si-TUFM, or si-TUFM plus TUFM-FLAG. Plasmids expressing the RNP-NP complex and the gene for CAT driven by the polymerase I gene promoter were cotransfected into 293T cells. At 48 h posttransfection, total protein lysates were extracted and used to detect levels of virus-like CAT reporter protein expression (which represents viral polymerase activity) by CAT-ELISA. The results shown are the mean ± the standard error of the mean of three independent experiments, and statistical significance was determined by one-way analysis of variance (ns, no significance). Validation of knockdown efficiency of siRNA and each component of the RNP-NP complex by Western blotting, with results shown at the bottom. Download FIG S7, TIF file, 0.6 MB. Copyright © 2017 Kuo et al. 2017 Kuo et al. This content is distributed under the terms of the Creative Commons Attribution 4.0 International license.

**FIG 4:**
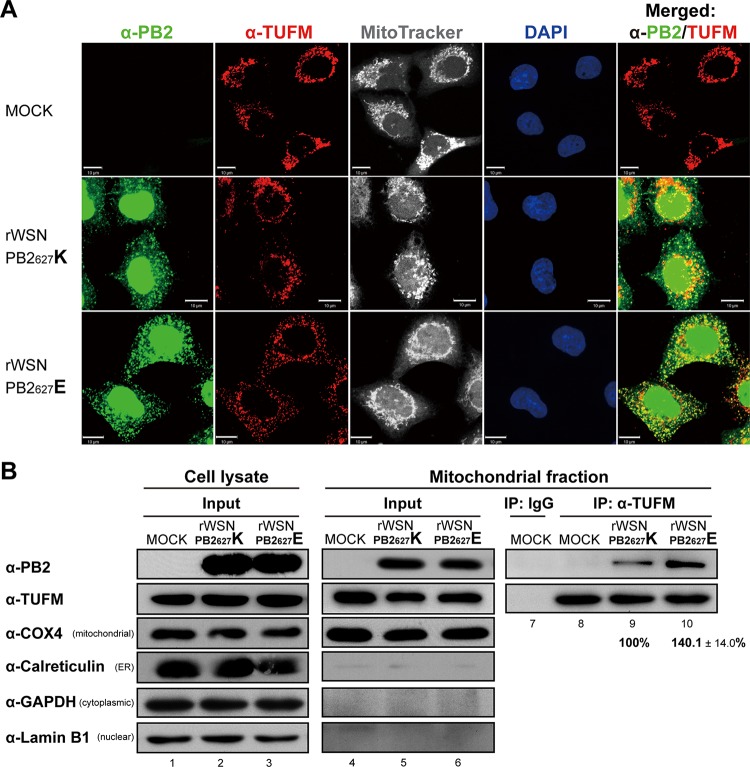
Subcellular localization and mitochondrial presence of TUFM-PB2 in infected human cells. (A) Subcellular localization of TUFM and PB2 in infected A549 cells was detected by IFA. A549 cells were infected with either rWSN PB2_627_K or rWSN PB2_627_E virus at an MOI of 10 for 9 h. Cells were probed with anti-PB2 and anti-TUFM antibodies, MitoTracker (as a mitochondrial marker), and DAPI (as a nuclear marker). The data are representative of three independent experiments. Scale bars, 10 μm. (B) Copurification of TUFM and PB2 in the mitochondrial fraction of infected 293A cells. Cell lysates (50 μg, lanes 1 to 3) were probed with anti-PB2, anti-TUFM, anti-COX4 (as a mitochondrial marker), anti-calreticulin (as an endoplasmic reticulum [ER] marker), anti-GAPDH (as a cytoplasmic marker), and anti-lamin B1 (as a nuclear marker) antibodies. Mitochondrial fractions used for input control (25 μg, lanes 4 to 6) prior to immunoprecipitation (IP) were also immunoblotted with the antibodies listed above. Fractions were subject to IP with either IgG (lane 7, as an NC) or anti-TUFM antibody (lanes 8 to 10). Immunoprecipitated mitochondrial fractions were then subjected to immunoblotting with anti-PB2 and anti-TUFM antibodies, and quantitative results of PB2 were divided by TUFM levels and are presented as the mean ± the standard error of the mean. Three independent experiments were performed.

The subcellular localization of TUFM and PB2 in cells infected with either rWSN PB2_627_K or rWSN PB2_627_E was examined by immunofluorescence assay (IFA). At 9 hpi at an MOI of 10, colocalization of TUFM with either PB2_627_K or PB2_627_E was detected in mitochondria (yellow spots in the merged images of Fig. 4A). No significant localization differences were observed between PB2_627_K and PB2_627_E, and TUFM signals were detected in mitochondria regardless of whether or not there was viral infection (Fig. 4A). Qualitative analysis of the interaction between TUFM and PB2 was subsequently performed via mitochondrial fractionation, followed by TUFM immunoprecipitation (TUFM-IP) in 293A cells. 293A cells were selected for this experiment because of their abundant mitochondrial fraction, their genetic background similar to that of the 293T cells used in the initial experiments of this study (Fig. 1 and 2), and their strong adherence to plastic dishes for facilitation of viral infection. Mitochondrial fractions were isolated from 293A cells infected with either rWSN PB2_627_K or rWSN PB2_627_E (Fig. 4B), and the mitochondrial marker COX4 was detected at equal levels in input controls (lanes 4 to 6 of Fig. 4B). Quantitative analysis of copurified TUFM-PB2_627_K and TUFM-PB2_627_E in mitochondrial fractions was performed, and TUFM-PB2_627_E levels were found to be 1.4-fold higher than TUFM-PB2_627_K levels (lanes 9 and 10 of Fig. 4B), suggesting that TUFM has higher levels of interaction with avian-signature PB2_627_E than with human-signature PB2_627_K in the mitochondria of infected human cells. Taken together, these results indicate selectively higher binding affinity for avian-signature PB2_627_E by TUFM in mitochondria (Fig. 4B), and this may be correlated with the inhibitory effect of TUFM on avian-signature influenza virus replication in human cells (Fig. 3).

### TUFM interactions with PB2_627_K and PB2_627_E do not differentially affect type I IFN promoter activity.

Previous research has reported that TUFM interacts with NLRX1, Atg12, Atg5, and Atg16L1 to form a molecular complex in mitochondria, and this complex not only enhances VSV-induced autophagy but also inhibits type I IFN expression by acting against RIG-I (23). Though NLRX1, Atg5, Atg12, and Atg16L1 were not listed among the 168 putative associated proteins that we identified as interacting with the WSN PB2_627_K and PB2_627_E viral proteins (see Table S1), immunoprecipitates of FLAG-tagged WSN PB2_627_K or PB2_627_E pulled down by FLAG-IP revealed that PB2 also binds to endogenous NLRX1 but probably not endogenous Atg5-Atg12 or endogenous Atg16L1 (see Fig. S8A). However, the amount of endogenous NLRX1 associated with PB2_627_K and PB2_627_E was, respectively, only 3.0% and 32.5% of the PB2-TUFM binding levels (see lanes 7 and 8 of Fig. S8A). We subsequently assessed whether the PB2-TUFM interaction competes with the TUFM-NLRX1 interaction and found that levels of exogenously overexpressed NLRX1 slightly decreased in response to increasing levels of PB2_627_E (see lanes 6 to 8 of Fig. S8B). The PB2-TUFM interaction therefore may compete with the interaction between TUFM and NLRX1. Interestingly, expression of TUFM-FLAG alone was able to reduce IFN promoter activity to 14% of that of cells exogenously expressing the caspase activation and recruitment domain (CARD) of RIG-I (see lane 3 of Fig. S8C), and this confirmed previous research showing that TUFM can inhibit IFN (23). Moreover, IFN promoter activity was, respectively, suppressed to 5.1% and 5.6% of that of cells exogenously expressing RIG-I following cotransfection of TUFM with PB2_627_K or PB2_627_E, with no significant difference (see lanes 5 and 7 of Fig. S8C).

10.1128/mBio.00481-17.8 FIG S8  WSN PB2_627_K-TUFM and PB2_627_E-TUFM interactions have no effect on IFN promoter activity. (A) Immunoprecipitates of FLAG-tagged WSN PB2_627_K or PB2_627_E were pulled down by a FLAG-IP procedure similar to that used for Fig. 1A and 2C and then subjected to immunoblotting with additional anti-NLRX1, anti-Atg12, and anti-Atg16L1 antibodies. NLRX1 protein bands were normalized to PB2_627_K-TUFM. (B) Immunoprecipitates of FLAG-tagged TUFM cotransfected with NLRX1-Myc, and 1, 3, and 5 μg of PB2_627_E-Myc plasmids (lanes 2 to 4 and 6 to 8) were pulled down by FLAG-IP. NLRX1 protein bands were normalized to each TUFM and then compared with the control (lane 5). (C) IFN promoter activity in 293T cells cotransfected with TUFM and PB2_627_K or PB2_627_E. Protein bands were quantitatively measured by ImageJ software. Data are the mean ± the standard error of the mean of three independent experiments. Statistical significance was determined by one-way analysis of variance. ***, *P* < 0.001; ns, no significance. Download FIG S8, TIF file, 0.4 MB. Copyright © 2017 Kuo et al. 2017 Kuo et al. This content is distributed under the terms of the Creative Commons Attribution 4.0 International license.

### TUFM-dependent autophagy may selectively influence avian-signature PB2_627_E virus replication.

**FIG 5:**
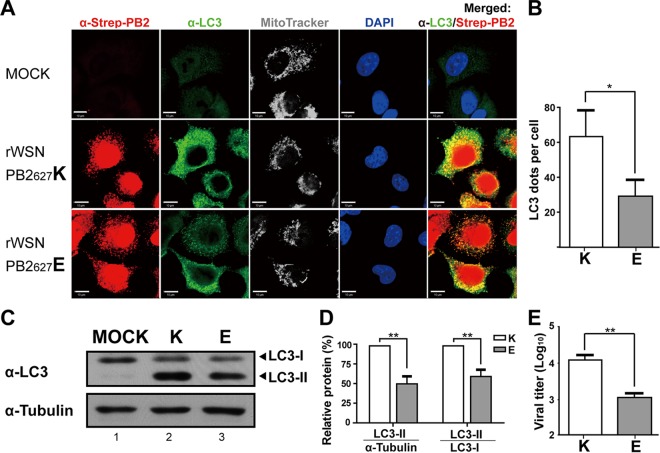
Levels of autophagy in human cells infected with the rWSN PB2_627_K and rWSN PB2_627_E viruses. (A) Subcellular localization of LC3 in influenza A virus-infected human cells as detected by IFA. A549 cells were infected with either rWSN PB2_627_K or rWSN PB2_627_E virus at an MOI of 10 for 9 h. Cells were probed with anti-Strep (represents PB2) and anti-LC3 (represents autophagy) antibodies, MitoTracker (represents TUFM), and DAPI (represents the nucleus). The data are representative of three independent experiments. Scale bars, 10 μm. (B) Quantitative analysis of LC3 punctate dots per cell. (C) Levels of LC3 in influenza A virus-infected human cells were detected by Western blotting. 293A cells were infected with either rWSN PB2_627_K or PB2_627_E virus at an MOI of 2 for 9 h. Protein lysates (50 μg) were probed with anti-LC3 and anti-α-tubulin antibodies. (D) Quantitative analysis of autophagy in influenza A virus-infected human cells. Protein bands (C) were quantitatively measured by ImageJ software. For quantitative analysis of LC3 in PB2_627_K versus PB2_627_E virus-induced autophagy in 293A cells, levels of LC3-II were divided by anti-tubulin or anti-LC3-I antibody and presented as percentages after normalization to the levels of PB2_627_K. (E) Viral titers of culture supernatant from rWSN PB2_627_K or rWSN PB2_627_E virus-infected 293A cells (lanes 2 and 3 of panel C), for which levels of autophagy were measured (C, D). Statistical analyses were conducted with GraphPad Prism 5. Data are the mean ± the standard error of the mean of three independent experiments. Statistical significance was determined by unpaired *t* tests. **, *P* < 0.01; *, *P* < 0.05.

As previous research indicated that TUFM can enhance autophagy in conjunction with reduction of type I IFN promoter activity in VSV-infected human cells (23), we also sought to ascertain whether TUFM inhibition of avian-signature PB2_627_E influenza A virus in human cells is associated with autophagy. The subcellular localization of PB2 and LC3, a specific marker of autophagosome formation, was examined in infected A549 cells by IFA. LC3 signals were detected in both rWSN PB2_627_K- and rWSN PB2_627_E-infected cells but were less visible in mock-infected controls (Fig. 5A). LC3 punctate dots (shown in green) were calculated per cell (Fig. 5B), and the results indicated that LC3 levels were lower in rWSN PB2_627_E-infected cells than in rWSN PB2_627_K-infected cells. Yellow dots represent colocalized PB2 and LC3 (right side of Fig. 5A) and show that autophagy occurred in both rWSN PB2_627_K- and rWSN PB2_627_E-infected cells. Quantitative analysis of LC3 was conducted by Western blotting of infected 293A cell lysates, with two forms of LC3 separated, cytoplasmic LC3-I and autophagic LC3-II associated with the autophagosome membrane. As the conversion of LC3-I to LC3-II is reflective of autophagic induction (30), LC3-II levels relative to those of α-tubulin controls were used to quantify autophagy levels. Both LC3-II/α-tubulin and LC3-II/LC3-I ratios in rWSN PB2_627_E-infected cells were 50.6% and 59.7% of those in rWSN PB2_627_K-infected cells (Fig. 5C and D), indicating that autophagy levels were lower in rWSN PB2_627_E-infected cells than in rWSN PB2_627_K-infected cells. rWSN PB2_627_E viral titers were almost 10-fold lower than rWSN PB2_627_K viral titers at 9 hpi with an MOI of 2 (Fig. 5E), which corroborates the fact that avian influenza A virus does not replicate efficiently in human cells.

**FIG 6:**
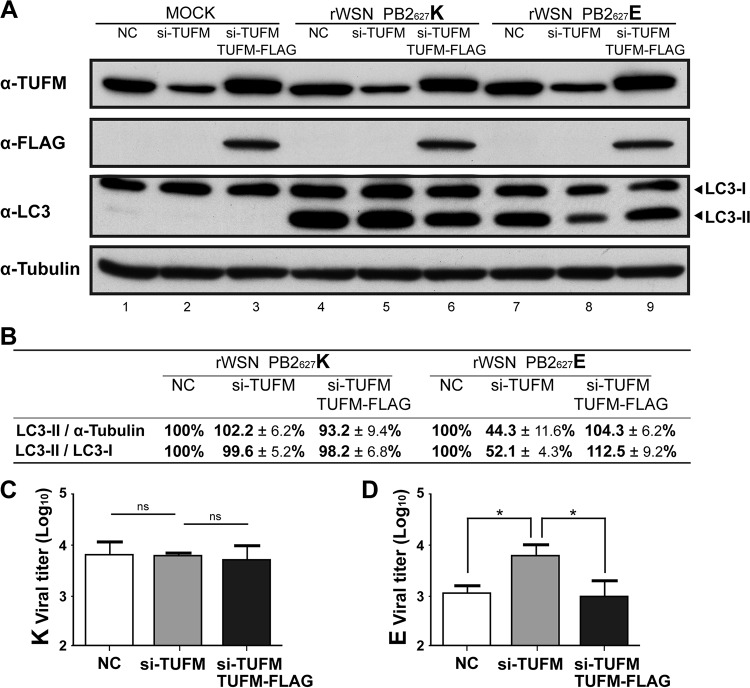
TUFM-dependent autophagy may selectively influence avian-signature PB2_627_E viral replication in infected human cells. (A) Effect of TUFM on autophagy in rWSN PB2_627_K or rWSN PB2_627_E virus-infected 293A cells transfected with NC siRNA, si-TUFM, or si-TUFM plus TUFM-FLAG plasmid. Protein lysates of infected cells (50 μg) were probed with anti-TUFM, anti-FLAG (represents TUFM-FLAG), anti-LC3, and anti-α-tubulin antibodies. (B) Quantitative analysis of TUFM-dependent autophagy in influenza A virus-infected human cells. Protein bands in panel A were quantitatively measured by ImageJ software. Levels of LC3-II were divided by anti-tubulin or anti-LC3-I antibody and are presented as percentages after normalization to the levels of NC siRNA. Viral titers of culture supernatants from 293A cells treated with NC siRNA, si-TUFM, or si-TUFM plus TUFM-FLAG and infected with either rWSN PB2_627_K (C) or rWSN PB2_627_E (D) virus at an MOI of 2 for 9 h (lanes 4 to 9 of panel A), in which TUFM-dependent autophagy was measured (A, B). Statistical analyses were conducted with GraphPad Prism 5. Data are the mean ± the standard error of the mean of three independent experiments. Statistical significance was determined by unpaired *t* tests. *, *P* < 0.05; ns, no significance.

Autophagy levels and viral titers remained constant with no significant difference in TUFM-deficient human cells infected with rWSN PB2_627_K virus (Fig. 6A to C). Interestingly, LC3-II/α-tubulin and LC3-II/LC3-I ratios were, respectively, reduced to 44.3% and 52.1% in TUFM-deficient 293A cells (treated with si-TUFM) infected with rWSN PB2_627_E, compared to infected cells treated with control siRNA. LC3-II/α-tubulin and LC3-II/LC3-I ratios were, respectively, restored to 104.3% and 112.5% of the control ratios in si-TUFM plus TUFM-FLAG transfected cells infected with rWSN PB2_627_E (Fig. 6A and B). rWSN PB2_627_E viral titers were increased 6.1-fold in TUFM-deficient cells at 9 hpi with an MOI of 2, while rWSN PB2_627_E viral titers were comparable to those of controls in si-TUFM plus TUFM-FLAG transfected cells (Fig. 6D; similar to Fig. 3E). The results indicate that TUFM-dependent autophagy was reduced in TUFM-deficient cells infected with PB2_627_E virus, and this correlated with an increase in viral titers (Fig. 6A, B, and D).

## DISCUSSION

**FIG 7:**
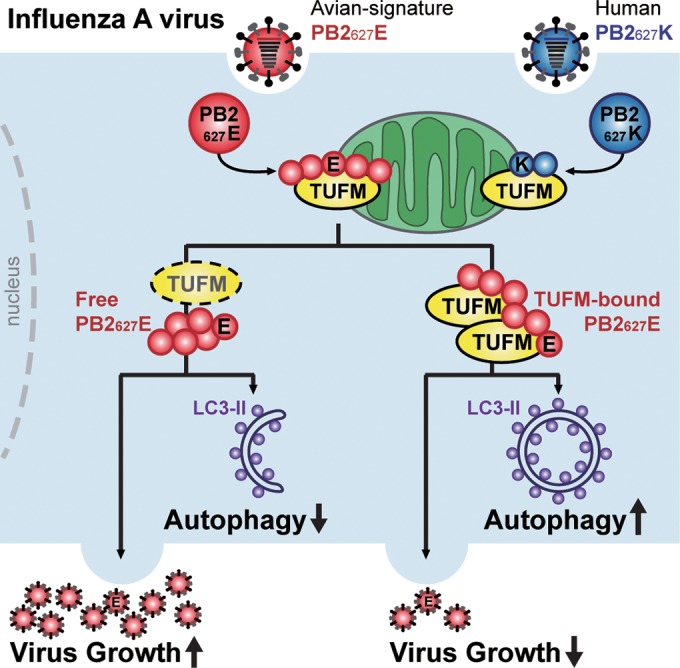
Proposed model for how TUFM-dependent autophagy selectively influences PB2_627_E viral replication in infected human cells. The model shows TUFM has a stronger interaction with avian-signature PB2_627_E than human-signature PB2_627_K in mitochondria, and therefore host factor TUFM may selectively interact in mitochondria with the PB2_627_E protein upon avian influenza virus infection. Binding of TUFM-PB2_627_E was impaired in TUFM-deficient human cells (indicated by a dotted ellipse), and this may free PB2_627_E to facilitate avian influenza viral replication, thus leading to observable increases in viral growth. However, with more TUFM-bound PB2_627_E, either free PB2_627_E is reduced or PB2_627_E ineffectively participates in viral replication, thereby reducing viral growth. In addition, TUFM promotes autophagy in avian-signature PB2_627_E-infected human cells, whereas TUFM deficiency decreased autophagy. This model proposes that TUFM-dependent autophagy selectively influences avian-signature PB2_627_E viral replication in infected human cells, and may serve as an intrinsic defense against avian influenza viruses.

Avian influenza A viruses generally do not replicate efficiently in human cells, and this was also observed in this study, as viral titers of rWSN PB2_627_E were almost 10-fold lower than those of rWSN PB2_627_K (Fig. 5E), suggesting that host restriction factors inhibit the viral replication of avian influenza virus in human cells. TUFM has a higher binding affinity for PB2_627_E than for PB2_627_K in both transfected human cells (Fig. 2C) and the mitochondrial fraction of virus-infected human cells (Fig. 4B). Accordingly, we propose a model (Fig. 7) in which human TUFM interacts with and binds avian-signature PB2_627_E of influenza virus in mitochondria. Binding between TUFM and PB2_627_E is impaired in TUFM-deficient human cells (indicated by a dotted ellipse in Fig. 7), and this may allow free PB2_627_E to facilitate influenza virus replication in human cells; as a result, rWSN PB2_627_E viral growth was observed to increase (Fig. 3B, E, and H). In contrast, in human cells expressing TUFM or in TUFM-deficient human cells reconstituted to express TUFM-FLAG, TUFM-bound PB2_627_E is more prevalent, and this may either reduce free PB2_627_E or disrupt its ability to participate in viral replication, thus leading to lower viral yields (Fig. 3). We therefore propose that TUFM can be considered a host restriction factor for the inhibition of avian influenza virus replication in human cells. In addition, TUFM has been reported to promote autophagy in VSV-infected cells (23), and this study also found that TUFM promoted autophagy in avian-signature PB2_627_E-infected human cells, whereas TUFM deficiency decreased autophagy (Fig. 6). This TUFM-dependent autophagy may serve as a defensive mechanism against avian influenza virus replication in human cells.

Although autophagy is known to act as a critical host defense mechanism against viral infection in cells (24, 25), influenza A virus is known to subvert autophagy for self-benefit. For example, influenza A viruses have been shown to reduce autophagy and maintain virion stability via direct interactions between viral matrix 2 protein and LC3 that block autophagosome fusion with lysosomes (26, 31); however, in this case, viral titers were not significantly affected (31). Another study showed that autophagy and lung inflammation were suppressed by 3-methyladenine (an autophagy inhibitor) or siRNA of autophagy-related genes in mouse lungs and A549 cells infected with avian influenza virus H5N1 carrying PB2_627_K, but viral titers were also not significantly altered (32). Similarly, viral titers in TUFM-deficient human cells infected with rWSN PB2_627_K were not significantly affected (Fig. 6C), even though autophagy levels of rWSN PB2_627_K virus remained consistently stronger than those of rWSN PB2_627_E virus (Fig. 6A and B and 5C and D). These results suggest that TUFM-dependent autophagy differs greatly between avian-signature PB2_627_E and human-signature PB2_627_K viruses and imply that differences in TUFM specificity can affect the host response to influenza virus infection.

Proteomic analysis (Fig. 1C and D; see Table S1) of PB2_627_K-specific proteins identified importin-α4 and importin-β1, which facilitate nuclear transport (12, 14), while common proteins included HSP90, which is involved in the assembly and nuclear import of the RNP complex (33); α-tubulin, which assists in transport of the RNP complex from the nucleus to the cytoplasmic membrane (34); and NPM1, NCL, and DDX3X, which are upregulated in both H1N1 and H5N1 influenza A virus-infected cells (12). This indicates that the current experimental design is effective and captures previously documented proteins.

Regarding the role of TUFM in other species during influenza A virus infections, mouse TUFM was previously found to be slightly upregulated at 72 hpi in mouse lung tissue infected with avian H9N2 PB2_627_E influenza A virus at 10^4^ PFU (35). A partial chTUFM protein sequence (352 aa; UniProt code P84172) identified by MS was previously released (36) and was found to have 68.5% similarity to human TUFM. A comparison of polymorphisms in aligned TUFM proteins and phylogenetic analysis suggests that different versions of TUFM exist in avian and mammalian species (see Fig. S1B to D). Therefore, TUFM-associated host restriction machinery for avian influenza viruses may be present only in mammalian species and not in avian species. The PB2 E627K substitution that facilitates human adaptation of avian influenza viruses is being detected with greater frequency in human samples since its possible inception in a single human host while remaining absent from chicken samples (37). This allows us to hypothesize that the key restriction factor(s) associated with PB2 must be absent from avian cells; otherwise, the PB2 E627K substitution that facilitates viral adaptation would appear at a greater frequency among avian species as well. This hypothesis may explain why TUFM inhibits avian-signature PB2_627_E viral replication specifically in human cells (Fig. 3) and not chicken cells (see Fig. S6).

In conclusion, we used a differential proteomic approach to identify a novel host restriction factor, TUFM, that preferentially interacts with avian-signature PB2_627_E in the mitochondria of human cells infected with avian-signature influenza A virus. TUFM impedes the replication of avian-signature influenza virus in human cells, possibly through mediation of autophagy. Our findings provide new insight into the role of mitochondria in the host defense against avian influenza viruses and may have important implications for future antiviral research.

## MATERIALS AND METHODS

### Cell lines.

HEK 293T (ATCC CRL-3216), HEK 293A (a subclone of the HEK 293 cell line that strongly adheres to plastic dishes; Invitrogen R70507), A549 (ATCC CCL-185), MDCK (ATCC PTA-6500), and DF-1 (ATCC CRL-12203) cell lines were cultivated in Dulbecco’s modified Eagle’s medium (DMEM; Gibco) containing 10% fetal bovine serum (FBS; Gibco). NHBE cells (ATCC PCS-300-010) were grown in bronchial epithelial cell growth medium (Lonza) containing growth factors, cytokines, and supplements. Primary chicken embryo fibroblast (CEF) cells were prepared from 9- to 11-day-old embryonated eggs. After removal of the head, limbs, viscera, and vertebrae, embryos were cut into 2- to 5-mm^3^ pieces and washed in phosphate-buffered saline. CEF cells were derived by trypsinization (0.25% trypsin) for 5 min at 37°C. After filtration, CEF cells were cultured in DMEM containing 10% FBS and antibiotics.

### Rapid amplification of cDNA 3′ ends.

First-round PCR was performed with a specific forward primer for chTUFM (TGCGAGTGAGGACGTCCAAGAT; nt −20 to 2; XM_015274224) and a reverse oligo(dT)-XbaKpnBam adaptor primer (CTGATCTAGAGGTACCGGATCCTTTTTTTTTTTTTTTTTT). Subsequently, a nested PCR was performed with a specific forward primer for chTUFM (TGGGCATGCTGACTACGTTAAG; nt 327 to 348; XM_015274224) and the XbaKpnBam reverse primer.

### Reverse genetics.

The Pol I and Pol II plasmids of the influenza A/WSN/1933(H1N1) virus were kindly provided by Robert G. Webster, Department of Infectious Diseases, St. Jude Children’s Research Hospital, Memphis, TN. To generate the recombinant viruses, 12 plasmids were cotransfected into 293T cells (38, 39) with Lipofectamine 2000 (Invitrogen) and supplemented with minimal essential medium (Gibco). At 18 to 20 h posttransfection, the transfection medium was replaced with DMEM without FBS. At 32 h posttransfection, the supernatant was collected and amplified in MDCK cells. Viral titers in MDCK cells were measured by a plaque assay.

### Plasmids and mutagenesis.

For FLAG-IP, the CDSs of PB2 proteins derived from the influenza A/WSN/1933(H1N1) and swine-origin influenza A/Taiwan/126/2009(pdmH1N1) viruses were cloned into vector pFLAG-CMV5.1 and the resulting plasmids were respectively named pFLAG-CMV5.1-WSN-PB2_627_K and pFLAG-CMV5.1-pdmH1N1-PB2_590_S/_591_R. The CDS of PB2 derived from A/Anhui/1/2013(H7N9) was synthesized (IDT) and cloned into the pFLAG-CMV5.1 vector to derive pFLAG-CMV5.1-H7N9-PB2_627_K. Site-directed mutagenesis was performed in accordance with the manufacturer’s instructions to generate plasmids pFLAG-CMV5.1-WSN-PB2_627_E, pFLAG-CMV5.1-pdmH1N1-PB2_590_G/_591_Q, pFLAG-CMV5.1-H7N9-PB2_627_E, and pFLAG-CMV5.1-H7N9-PB2-D_701_N. For reverse genetics, the polI-WSN-PB2_627_K-Cstrep plasmid (a gift from Nadia Naffakh, Institut Pasteur, Paris, France) was used to generate plasmid polI-WSN-PB2_627_E-Cstrep. For overexpression, the CDS of human TUFM from 293T cells was cloned into vectors pFLAG-CMV5.1 and pcDNA3.1/myc-HisA and named TUFM-FLAG and TUFM-Myc, respectively; the CDS of chTUFM from CEF cells was cloned into vector pcDNA3.1/myc-HisA and named chTUFM-Myc. For domain mapping, domain truncation mutants of human or chicken TUFM were generated with the Q5 site-directed mutagenesis kit (New England Biolabs) in accordance with the manufacturer’s instructions. For the polymerase activity assay, pPOLI-CAT-RT was provided by George Brownlee (Sir William Dunn School of Pathology, Oxford, United Kingdom), and the CDSs of PB1, PA, and NP derived from pdmH1N1 were, respectively, cloned into pcDNA3 expression vectors. The CDS of NLRX1 was subcloned from pENTER-NLRX1-Flag-His (ViGene) into the pcDNA3.1/myc-HisA vector and named NLRX1-Myc.

### Viruses.

The WSN-Cstrep recombinant virus (a generous gift from Nadia Naffakh, Institut Pasteur, Paris, France) (40) was termed the rWSN PB2_627_K virus, while the WSN-Cstrep recombinant virus with a K627E substitution generated by reverse genetics was termed the rWSN PB2_627_E virus. MDCK and DF-1 cells were used to amplify rWSN PB2_627_K and rWSN PB2_627_E viruses, respectively. Viruses were titrated by plaque formation assay with monolayer MDCK cells. Direct sequencing of PCR products demonstrated that PB2 proteins derived from the rWSN PB2_627_K and rWSN PB2_627_E viruses were unchanged after multiple passages.

### FLAG-IP.

293T cells were transfected with either FLAG-tagged WSN PB2_627_K or FLAG-tagged WSN PB2_627_E. At 48 h posttransfection, total cell lysates were treated with the lysis buffer provided in the FLAG-IP kit (Sigma) for 30 min at room temperature. Protein samples (2 mg) were immunoprecipitated overnight at 4°C with the anti-FLAG M2 affinity gel. Interacting proteins were allowed to compete with 3×FLAG peptides for 30 min at 4°C, and the interaction between PB2 and cellular proteins was probed with antibodies.

### Myc-IP.

DF-1 cells were cotransfected with either Myc-tagged chicken or human TUFM and the FLAG-tagged WSN PB2_627_K or PB2_627_E plasmid. At 48 h posttransfection, total cell lysates were treated for 30 min at room temperature with the lysis buffer provided in the Myc-IP kit (Sigma). Protein samples (2 mg) were immunoprecipitated in accordance with the manufacturer’s instructions, and the interaction between Myc-tagged TUFM and PB2 was probed with antibodies.

### MS analysis.

The immunoprecipitates were separated with an 8 to 16% gradient gel and subjected to silver staining. Selected proteins were numbered, excised from the gel, extracted by in-gel digestion, and further identified by MALDI-TOF MS analysis. Accession numbers, protein names, MASCOT scores, and sequence coverage percentages of putative interacting proteins were evaluated by carbamidomethyl-fixed modification and oxidation-variable modification of the MASCOT database.

### Network analysis of protein-protein interactions.

Designations of genes for PB2_627_K-associated and PB2_627_E-associated proteins were used to query the STRING database (version 10.0) (41). The required confidence (score) was set at “high confidence.”

### Enrichment analysis of biological processes for functional annotation clustering analysis.

Designations of genes for common, PB2_627_K-specific, and PB2_627_E-specific proteins were used to query the functional annotation category GOTERM_MF_FAT clustering tool of the Database for Annotation, Visualization, and Integrated Discovery (DAVID) software v 6.7 (42).

### Protein modeling.

A homology model of the PB2 CTD for the influenza A/Anhui/1/2013(H7N9) virus was based on the A/Vietnam/1203/2004(H5N1) virus (PDB ID 3KC6) and constructed as previously described (29).

### TUFM-deficient cells.

ON-TARGETplus human TUFM siRNA (target sequence, CAGCUUCCCUUGCGUUUAA; 3′ untranslated region; nt −18 to −36; GE Healthcare) was termed si-TUFM. Two chTUFM siRNAs (target sequences, CCGCCAUCACCAAAGUGCUGUCGGA and CCGACUGCCCUGGGCAUGCUGACUA; nt 167 to 191 and 317 to 341; GeneDireX) were referred to as si-chTUFM-1 and si-chTUFM-2, respectively. 293T or 293A cells were transfected with 20 nM si-TUFM and Lipofectamine RNAiMAX (Invitrogen). A549 cells were transfected with 20 nM si-TUFM, with X-tremeGENE HP (Roche). NHBE cells were nucleofected with 30 nM si-TUFM by using the P3 Primary Cell 4D-Nucleofector X kit (Lonza) and the DC-100 program of the Amaxa 4D-Nucleofector device (Lonza). DF-1 cells were transfected with 100 nM si-chTUFM-1 or si-chTUFM-2 by using Lipofectamine RNAiMAX. At 18 to 24 h posttransfection with siRNA, cells were reseeded equally for further experiments such as plasmid transfection, virus infection, validation of knockdown efficiency, MTT assay, etc. Transfection efficiency was validated with Fluorescent Oligo (Invitrogen) for siRNA transfection, and protein expression of TUFM-deficient cells was validated by Western blotting.

### Establishment of TUFM +/− KO MDCK cells via CRISPR/Cas9-mediated genome editing.

Canine TUFM +/− KO MDCK cells (selected for being one of the cell lines approved by the U.S. Food and Drug Administration for vaccine development) were generated through optimized single-guide RNA (sgRNA; canine-targeting TUFM, accession no. NC_006588) and Cas9 expression pRGEN-Cas9-CMV plasmids (ToolGen). The third exon of canine TUFM was selected for sgRNA design. The sgRNA was located behind a U6 promoter and contained the target sequence TCCTCGGGCTCGTTCTTCAG**GGG** for canine TUFM genomic DNAs (the bold nucleotides are a protospacer-adjacent motif sequence not included in the sgRNA but recognized by the Cas9 protein). After transfection, MDCK cells were treated with hygromycin at 150 μg/ml for 2 days. Surviving cells were reseeded at 0.4 cells/well in a 96-well plate for the isolation of single cell clones. A stable canine TUFM heterozygotic +/− KO MDCK cell line was confirmed by fluorescent (F)-PCR and sequencing.

### Viral growth kinetics.

At 24 h posttransfection, TUFM-deficient human cells were reseeded in a six-well plate at 8 × 10^5^ cells per well. At 48 h posttransfection, cells were infected with rWSN PB2_627_K or rWSN PB2_627_E virus at an MOI of 0.001 for 12, 24, 36, 48, 60, or 72 h or at an MOI of 2 for 3, 6, 9, or 12 h. At each time point, viral supernatant was collected to determine viral titers via plaque assay.

### Western blotting and antibodies.

Precipitated or cell lysate proteins were separated by 8 to 12% SDS-PAGE, and samples were then transferred to polyvinylidene difluoride membranes (GE Health). Subsequently, these proteins were detected with the following antibodies: anti-FLAG-M2 (Sigma; 0.5 mg/ml; 1:1,000), anti-PB2 (Santa Cruz; 1:500), anti-TUFM (Santa Cruz; 1:800), anti-actin (Millipore; 1:4,000), anti-COX4 (GeneTex; 1:3,000), anti-calreticulin (GeneTex; 1:1,500), anti-glyceraldehyde-3-phosphate dehydrogenase (GAPDH; Abnova; 1:5,000), anti-lamin B1 (Abcam, Inc.; 1:4,000), anti-LC3B (Sigma; 1:2,500), anti-α-tubulin (Abcam, Inc.; 1:6,000), anti-Myc (Sigma; 1:1,500), anti-PB1 (GeneTex; 1:2,500), anti-PA (GeneTex; 1:2,000), anti-NP (generated by our colleague Cheng-Kai Chang; 1:10,000), anti-NLRX1 (Millipore; 1:100), anti-Atg12 (GeneTex; 1:1,500), and anti-Atg16L1 (GeneTex; 1:1,500).

### qRT-PCR.

Because anti-TUFM antibody was incapable of recognizing chTUFM expressed in DF-1 cells, the knockdown efficiency of si-chTUFM-1 and si-chTUFM-2 was evaluated by quantitative reverse transcription (qRT)-PCR instead of Western blotting. The first-strand cDNA synthesis was reverse transcribed with oligo(dT) and then subjected to qPCR with specific forward (TGGGCATGCTGACTACGTTAAG; nt 327 to 348) and reverse (TTCACATACACCACCACGTGC; nt 465 to 485) primers of chTUFM. Reactions were prepared with SYBR fast qPCR master mix (KAPA). PCR was performed on the LightCycler 480 (Roche) in the default run mode with SYBR green I. Chicken GAPDH (primers CATCATCCCAGCGTCCA and AGCACCCGCATCAAAGG; 283 bp) was used as a reference.

### MTT assay.

The viability of TUFM-deficient cells was validated by the MTT assay (Millipore) in accordance with the manufacturer’s instructions.

### CAT-ELISA.

RNP activity was measured with a chloramphenicol acetyltransferase enzyme-linked immunosorbent assay (CAT-ELISA; Roche) in accordance with the manufacturer’s instructions (29).

### IFA, antibodies, and confocal microscopy.

Mitochondria were stained with MitoTracker Red CMXRos (Invitrogen; 100 nM) for 15 min at room temperature before fixation with 4% paraformaldehyde for 15 min at room temperature. Subsequently, cells were permeabilized with 0.5% Triton X-100 for 15 min at room temperature and then probed with anti-PB2 (GeneTex; 1:500), anti-TUFM (Sigma; 1:500), anti-Strep (GenScript; 0.5 mg/ml; 1:500), and anti-LC3B (Sigma; 1:200) antibodies. Nuclei were visualized with ProLong Gold antifade reagent with 4',6-diamidino-2-phenylindole (DAPI; Invitrogen). All images were taken at ×1,000 magnification with a Zeiss LSM 510 Meta confocal microscope.

### Mitochondrial fractionation followed by TUFM-IP.

293A cells were infected with either rWSN PB2_627_K or rWSN PB2_627_E virus at an MOI of 2 for 9 h. Mitochondria were isolated from 293A cells with a mitochondrial/cytosol fractionation kit (Millipore) in accordance with the manufacturer’s instructions. Anti-TUFM antibody (3 μg; GeneTex) was coupled to protein G-Sepharose beads (GE Healthcare) and then incubated with 1 mg of the mitochondrial fraction in accordance with the manufacturer’s instructions. At least ten 15-cm dishes of 293A cells was collected for one reaction.

### IFN promoter activity assay.

A firefly luciferase reporter plasmid containing an IFN-β promoter (a gift from Michael Gale, University of Washington, Seattle, WA, USA), a pRL-TK *Renilla* luciferase control plasmid (for normalization of transfection efficiency), and a Myc-tagged RIG-I-CARD-expressing plasmid (N-RIG-I-Myc; provided by Helene M. Liu, National Taiwan University, Taiwan) were cotransfected into 293T cells in combination with a TUFM-FLAG, WSN PB2_627_K, or WSN PB2_627_E plasmid. At 24 h posttransfection, the firefly and *Renilla* luciferase activities of transfected cells were determined with a dual-luciferase reporter assay (Promega) in accordance with the manufacturer’s instructions. The normalized firefly luciferase activity represented the promoter activity of IFN-β.

### Nucleotide sequence accession number(s).

The full-length cDNA sequence of chTUFM, as derived from CEF cells, has been deposited in GenBank under accession number KY769204. The proteomic data set was deposited in the IMEx database (IntAct, IM-25425) (43).
