# Supplementary figures and images for: Inhibition of Avian Influenza A Virus Replication in Human Cells by Host Restriction Factor TUFM Is Correlated with Autophagy
Source: mBio. 2017 Jun 13;8(3):e00481-17. doi: 10.1128/mBio.00481-17 (PMC5472184; doi:10.1128/mBio.00481-17)

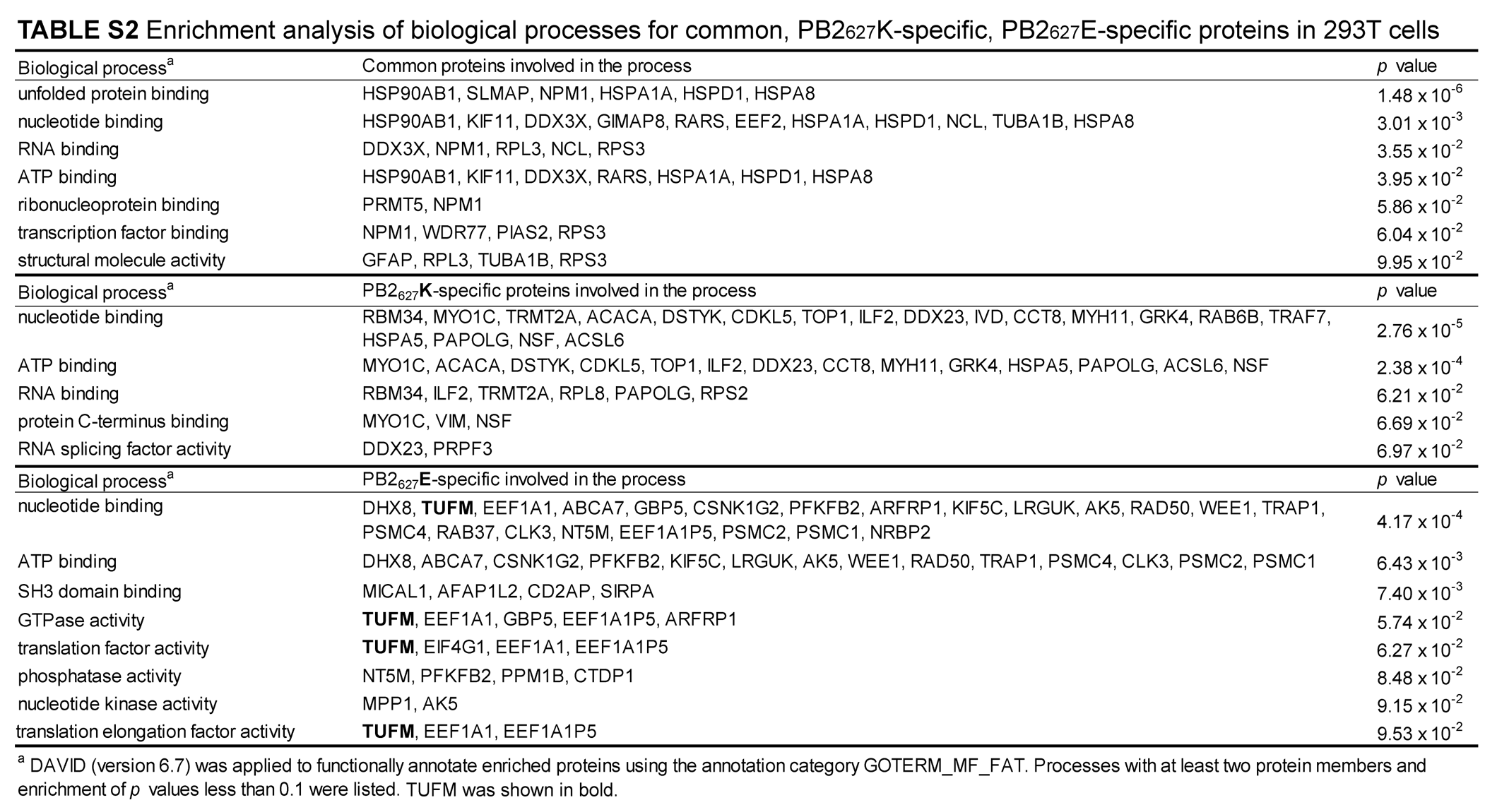

Supplement: TABLE S2 [file mbo003173342st2.tif]

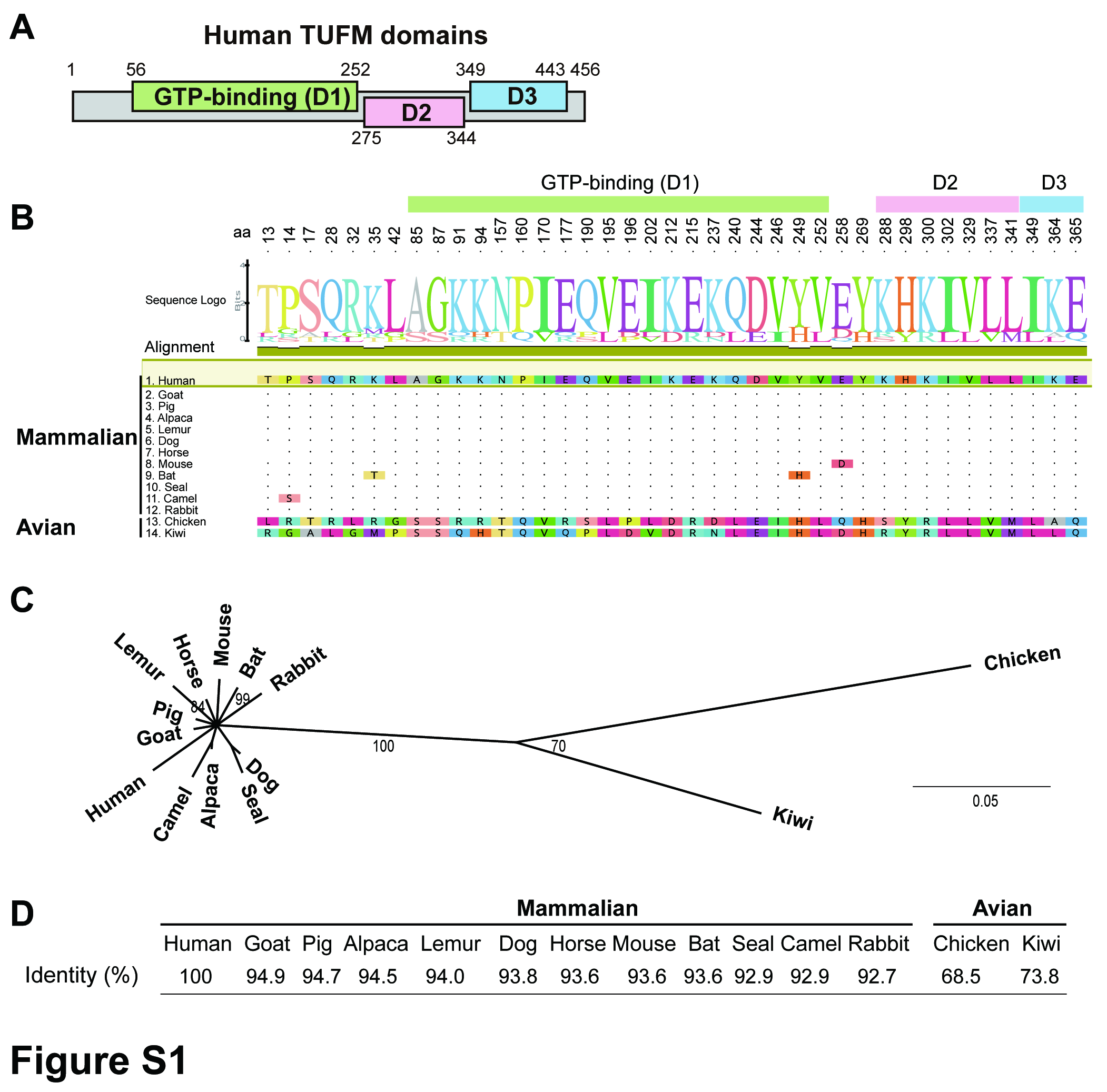

Supplement: FIG S1 [file mbo003173342sf1.tif]

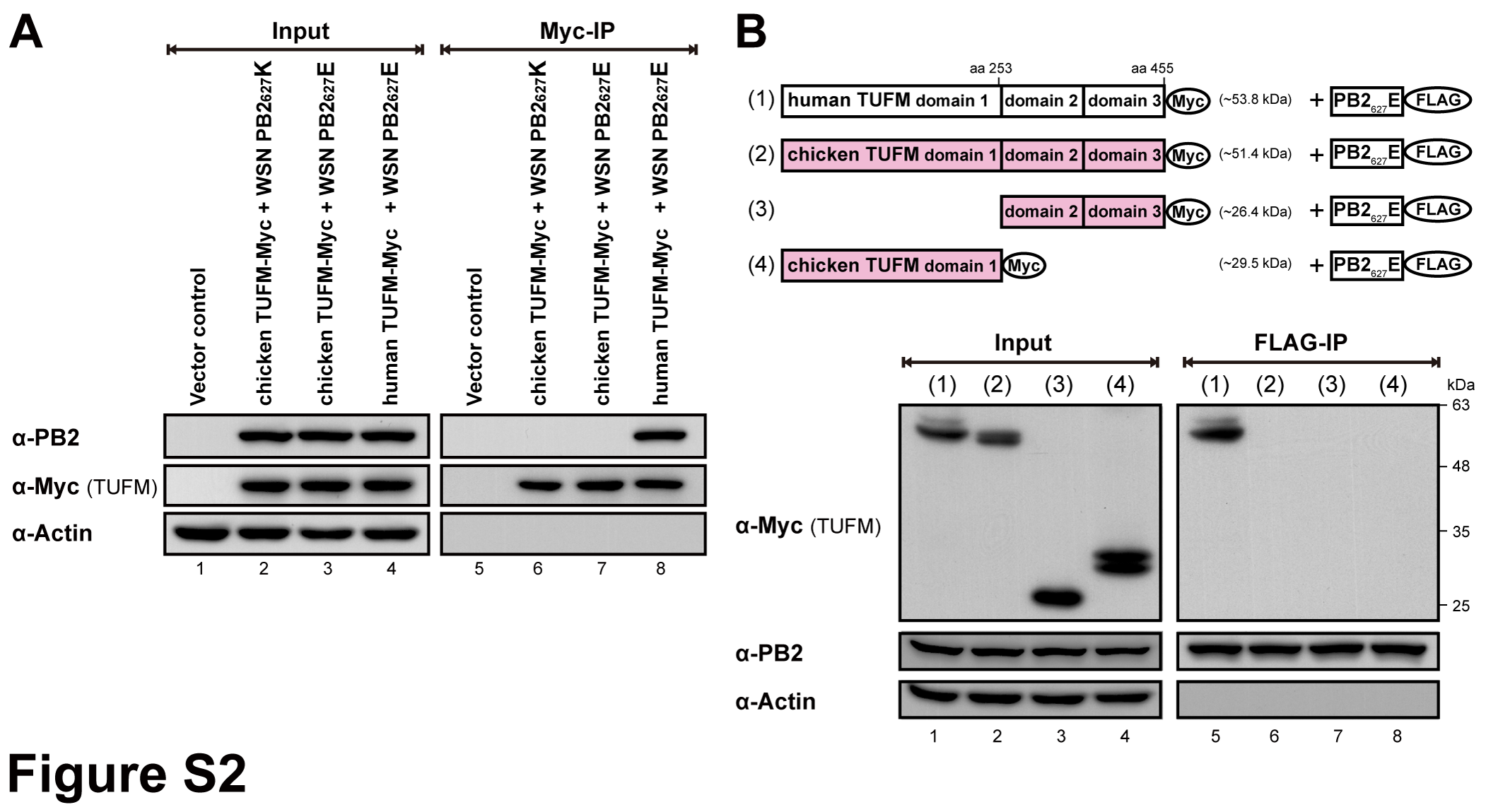

Supplement: FIG S2 [file mbo003173342sf2.tif]

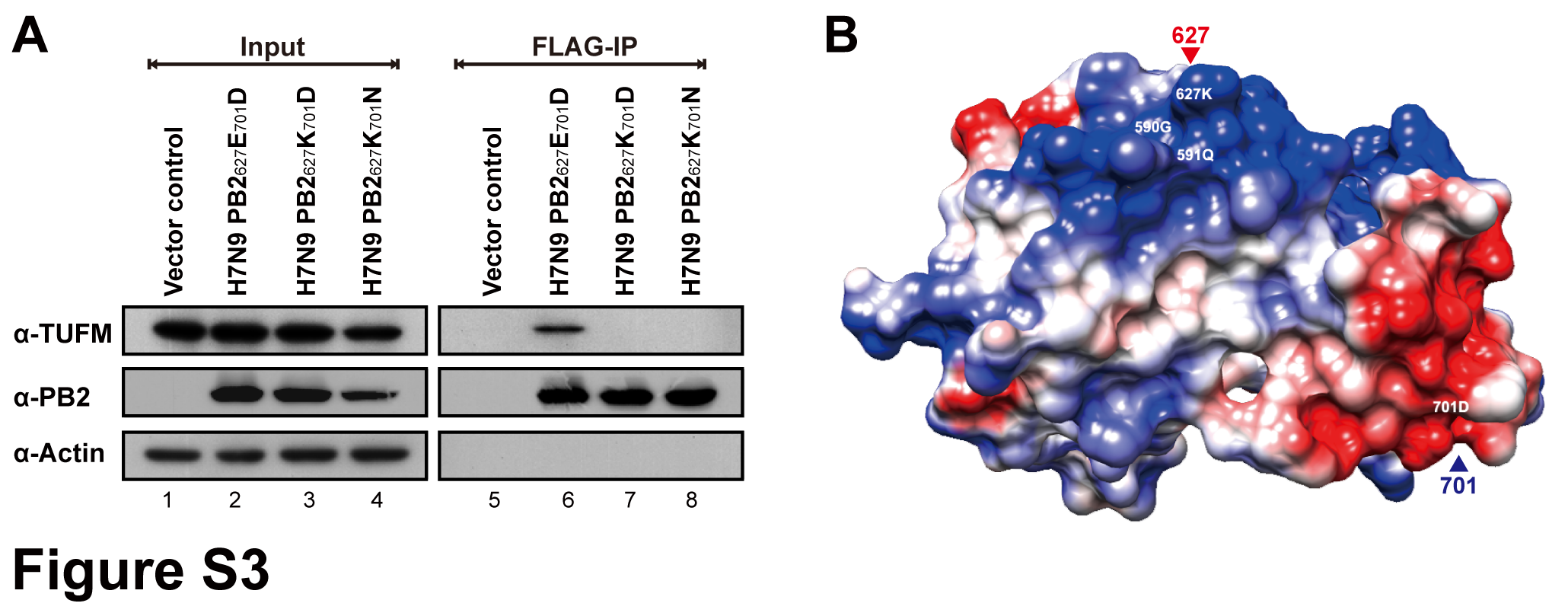

Supplement: FIG S3 [file mbo003173342sf3.tif]

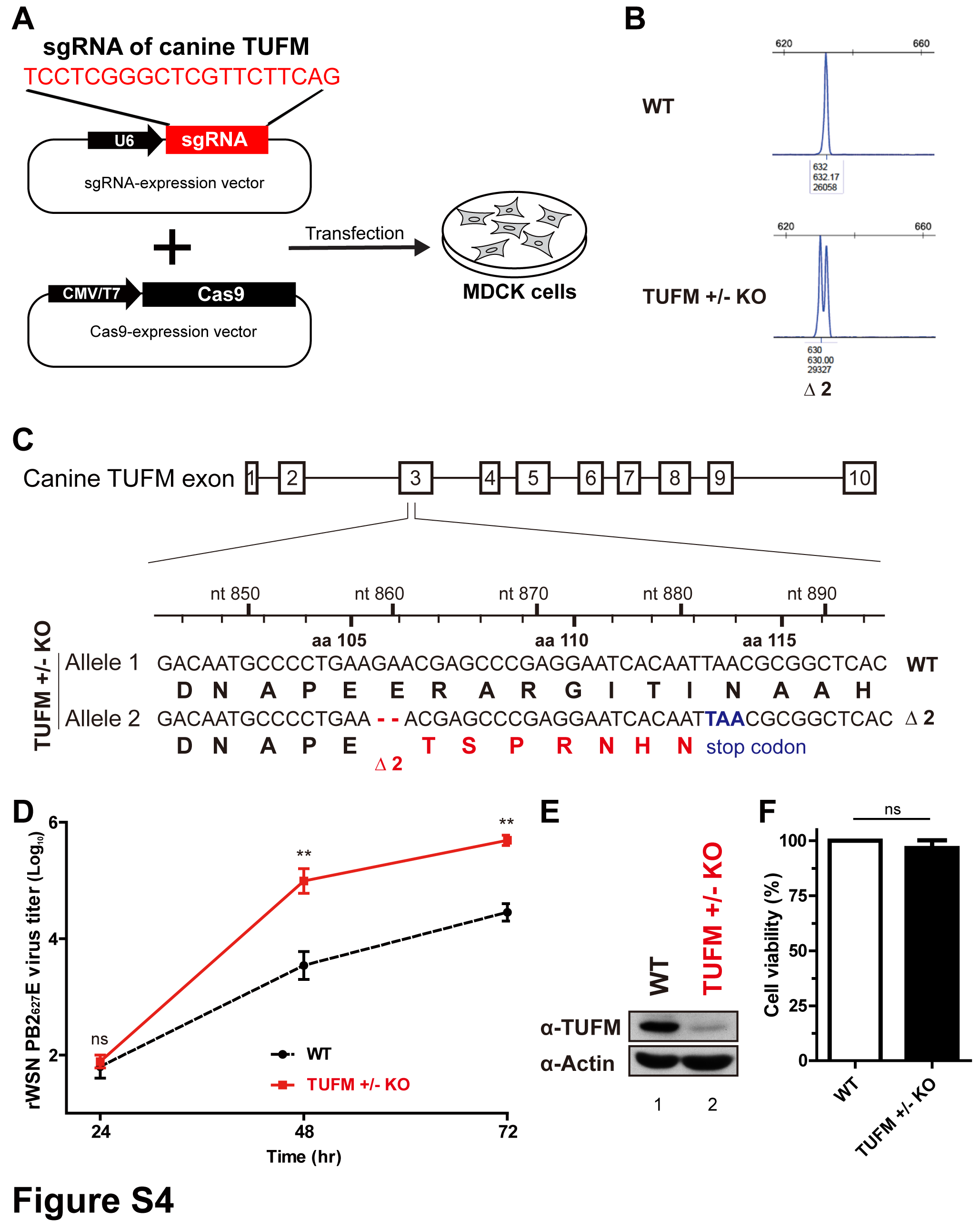

Supplement: FIG S4 [file mbo003173342sf4.tif]

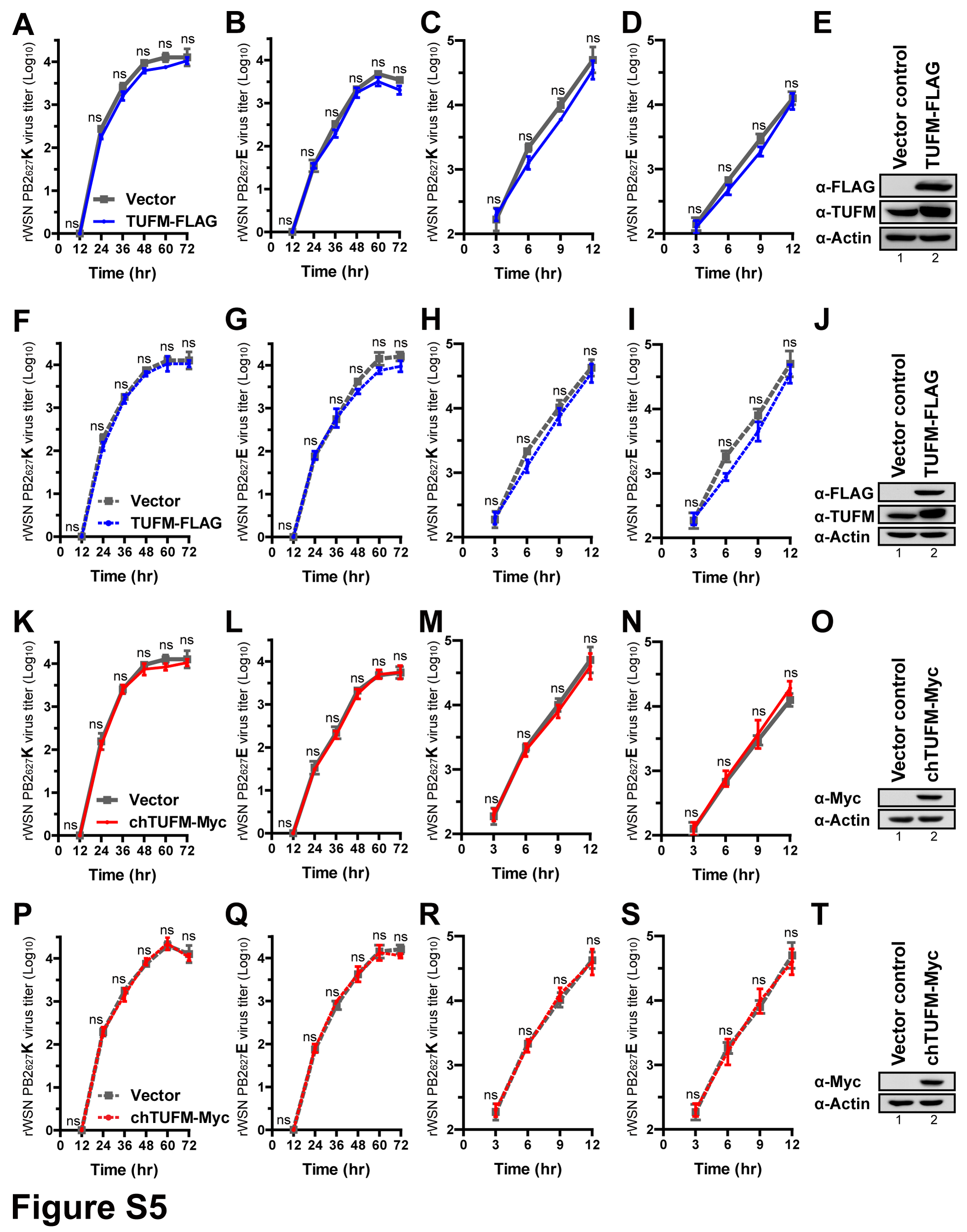

Supplement: FIG S5 [file mbo003173342sf5.tif]

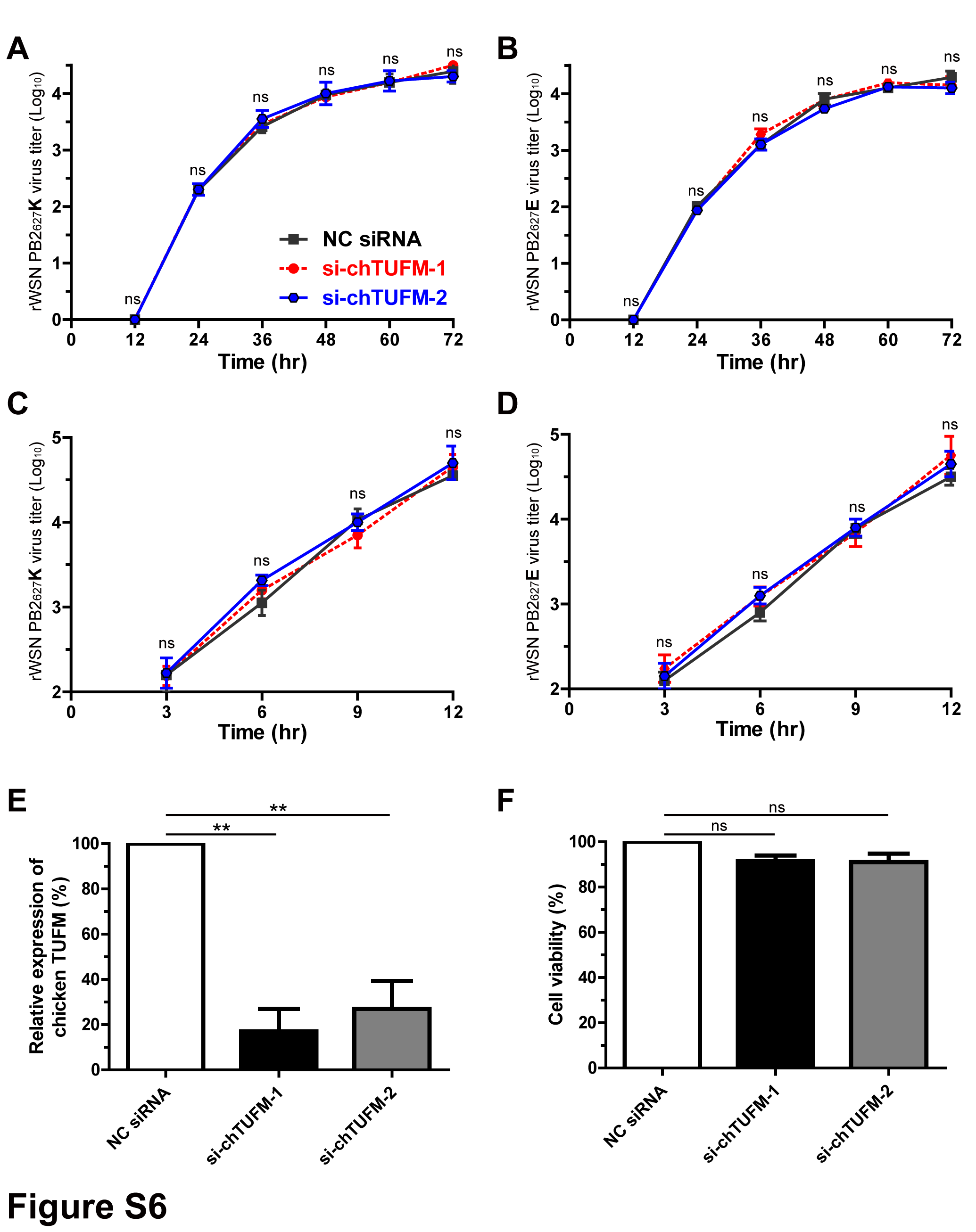

Supplement: FIG S6 [file mbo003173342sf6.tif]

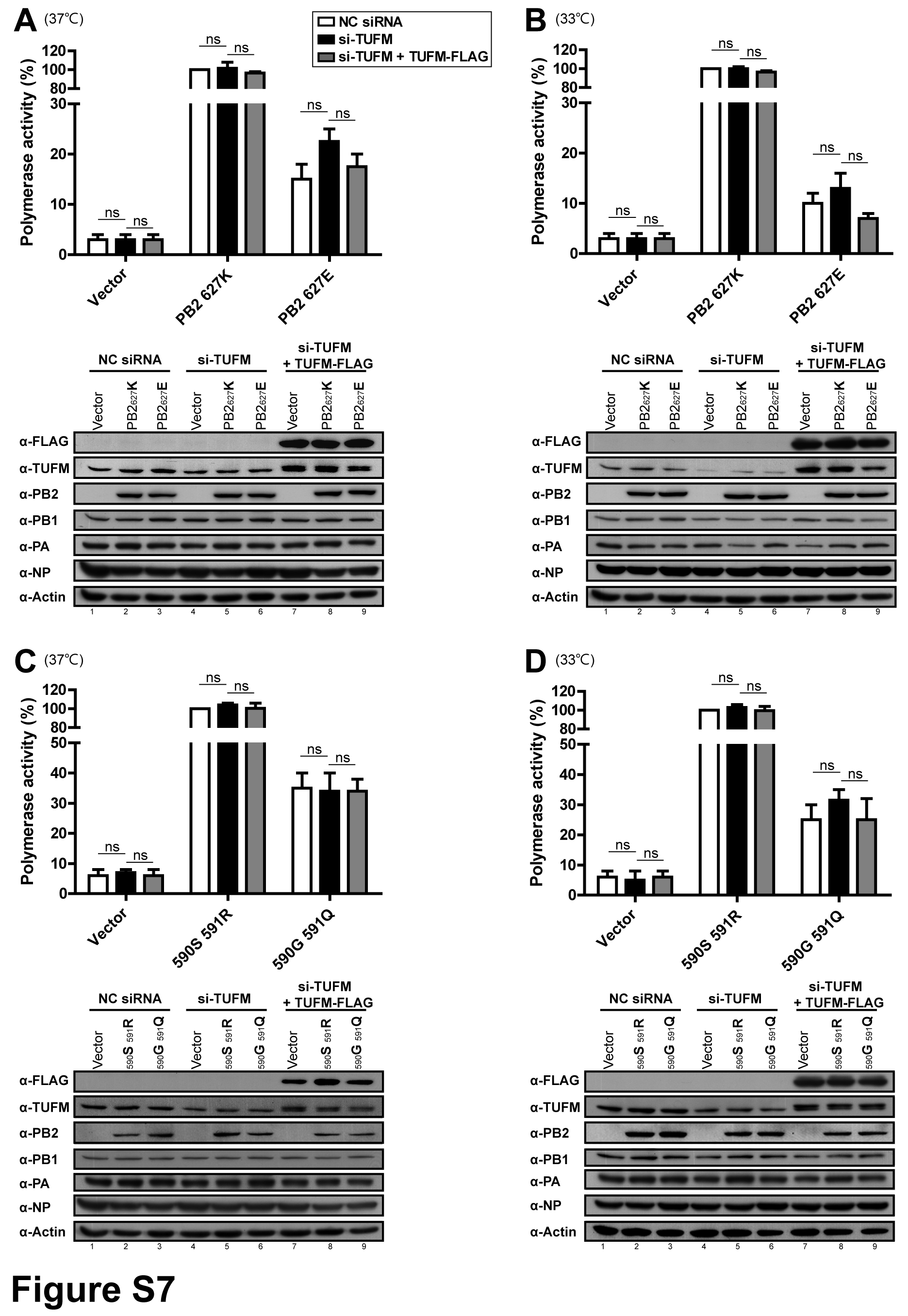

Supplement: FIG S7 [file mbo003173342sf7.tif]

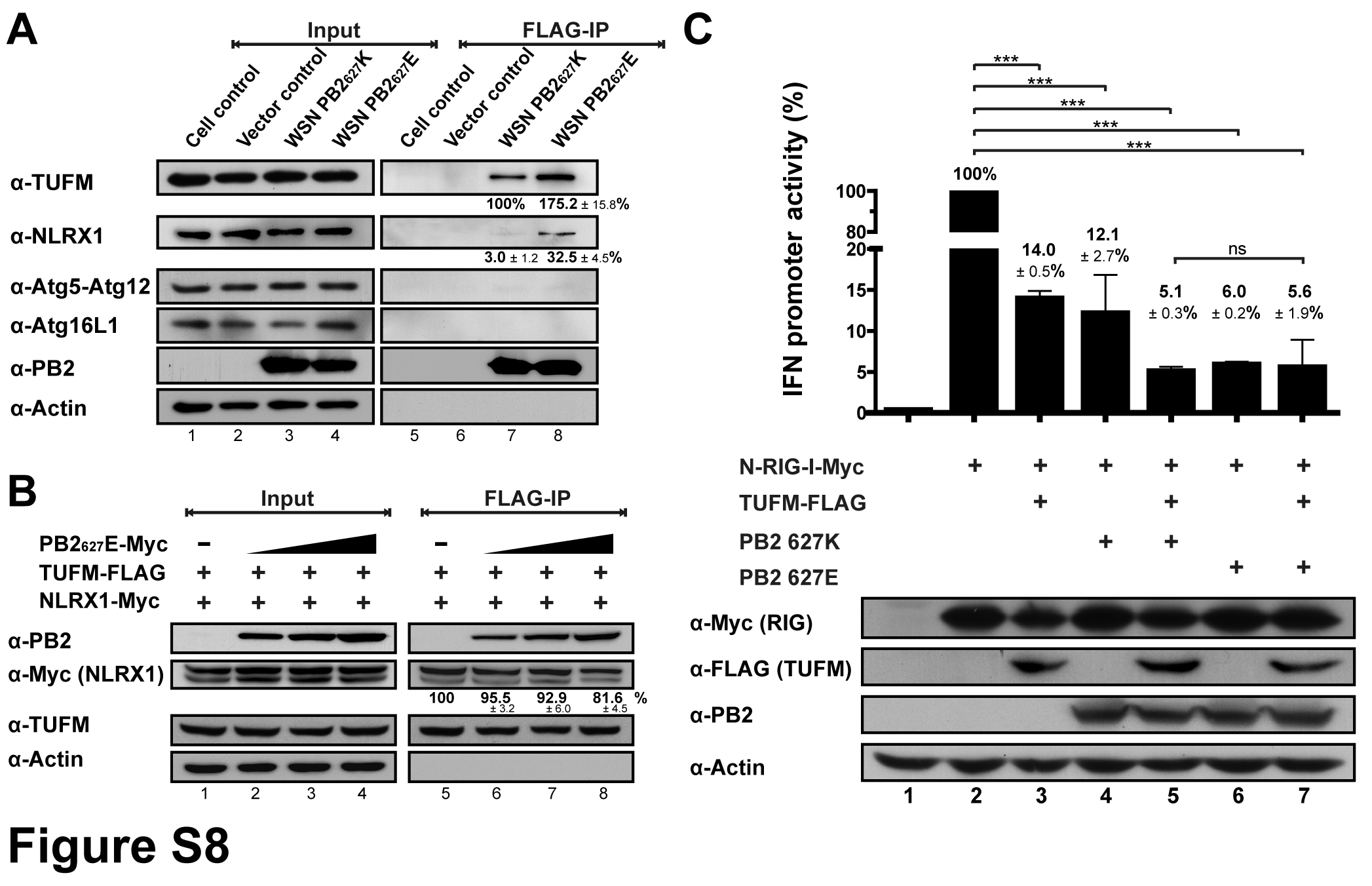

Supplement: FIG S8 [file mbo003173342sf8.tif]
